# Intracellular Oxidant Levels Are Crucial for Cell Survival and JAK/STAT Signaling in Classical Hodgkin’s Lymphoma

**DOI:** 10.3390/antiox15010090

**Published:** 2026-01-09

**Authors:** Julia Wildfeuer, Rashmi P. Dheenadayalan, Svenja Hartung, Malena Zahn, Timo P. Albrecht, Zhouli Cao, Alexey Ushmorov, Peter Möller, Nadine T. Gaisa, Ralf Marienfeld

**Affiliations:** 1 Institute of Pathology, Ulm University, 89081 Ulm, Germany; julia-1.wildfeuer@uni-ulm.de (J.W.); rashmi.dheenadayalan@uni-ulm.de (R.P.D.); svenja.hartung@uniklinik-ulm.de (S.H.); malena.zahn@r-pharm.com (M.Z.); timo_albrecht1@web.de (T.P.A.); peter.moeller@uni-ulm.de (P.M.); nadine.gaisa@uniklinik-ulm.de (N.T.G.); 2 Department of Dermatology and Allergic Diseases, Ulm University, 89081 Ulm, Germany; zhouli.cao@uni-ulm.de; 3 Institute of Physiological Chemistry, Ulm University, 89081 Ulm, Germany; alexey.ushmorov@uni-ulm.de

**Keywords:** classical Hodgkin’s lymphoma, JAK/STAT, PTP1B, NOX, reactive oxygen species, oxidant, apocynin, diphenylene iodonium, butylated hydroxyanisole

## Abstract

Although oxidants are known to be deleterious for cellular homeostasis by oxidizing macromolecules like DNA or proteins, they are also involved in signaling processes essential for cellular proliferation and survival. Here, we investigated the role of superoxide anion (O_2_^−^) and hydrogen peroxide (H_2_O_2_) homeostasis for the proliferation and survival of classical Hodgkin’s lymphoma (cHL) cell lines. Inhibition of NADPH oxidases (NOX) using apocynin (Apo) and diphenylene iodonium (DPI), or treatment with the antioxidant butylated hydroxyanisole (BHA), significantly reduced proliferation and induced apoptosis in HL cell lines. These effects correlated with transcriptomic alterations involving redox regulation, immune signaling, and cell cycle control. Interestingly, treatment with DPI or antioxidants attenuated constitutive Signal Transducer and Activator of Transcription (STAT) activity, as seen by decreased phospho-STAT6 levels and reduced STAT6 DNA binding. This suggests a sensitivity of the Janus kinase (JAK)/STAT pathway in cHL cell lines to O_2_^−^ and H_2_O_2_ depletion. Functional assays confirmed this by demonstrating partial restoration of proliferation or apoptosis in L428 cells that expressed constitutively active STAT6 or were transfected with small interfering RNAs (siRNAs) that targeted STAT regulators. These findings highlight that oxidants, particularly H_2_O_2_, act as both general oxidative stressors and essential modulators of oncogenic signaling pathways. Specifically, maintenance of oxidant homeostasis is critical for sustaining JAK/STAT-mediated growth and survival programs in cHL cells. Targeting redox homeostasis might offer a promising therapeutic strategy to impair JAK/STAT-driven proliferation and survival in cHL.

## 1. Introduction

Classical Hodgkin’s lymphoma (cHL) is one of the most frequent forms of B cell-derived lymphoma, with an incidence of about three cases per 100,000 persons per year. Current treatment protocols for cHL involve multiagent chemotherapy and/or radiotherapy, with a cure rate of about 80%. Relapses are usually treated with high-dose chemotherapy, radiation therapy, and autologous stem cell transplantation. However, challenges remain in a few patients who continue to progress after disease relapses [1,2].

The neoplastic component of cHL is the multinucleated Hodgkin’s Reed Sternberg (HRS) cell. Secretion of cytokines and chemokines by HRS cells leads to the recruitment and activation of various cell types into the tumor, including macrophages, dendritic cells, and lymphoid cells, thus creating an inflammatory environment. This inflammatory environment promotes the survival of the HRS cells by expressing additional cytokines, leading to the constitutive activation of various signaling pathways [3,4]. The Janus kinase/signal transducer and activator of transcription (JAK/STAT) signaling pathway is one of the major pathways essential for cHL and is constitutively activated in HRS cells. Under physiological conditions, JAK/STAT signaling is induced upon ligand binding of various cytokine and growth factor receptors, leading to the recruitment and activation of JAKs via trans-autophosphorylation. Activated JAKs phosphorylate STATs, leading to their dimerization, nuclear translocation, and induction of STAT target genes [5]. STAT6 plays a central role in TH2-associated immune regulation and the tumor microenvironment, and its dysregulation is implicated in several lymphoma subtypes including Hodgkin’s lymphoma, highlighting its relevance in cHL biology [6].

Oxidants, including superoxide anion (O_2_•^−^), hydrogen peroxide (H_2_O_2_), and the hydroxyl radical (•OH), were previously known for their microbicidal activity. More recently, it has been reported that oxidants are involved in the regulation of the cell cycle progression, apoptosis, and aging, pointing to a fundamental role of oxidants in tumor biology, which is highlighted by the elevated oxidants levels in almost all cancers [7]. Following the reduction of cellular oxidants levels in EBV-positive Burkitt’s lymphoma cells, for example, a G2-M phase cell cycle arrest was observed, leading to significant apoptosis [8]. Mechanistically, production of oxidants by NADPH-dependent oxidases (NOX) upon cytokine receptor engagement has been shown to alter the activity of several signaling pathways, including JAK/STAT [7,8,9,10]. For instance, a decrease in STAT5 phosphorylation was observed in NOX2-deficient naïve CD4+ T-cells, suggesting the role of NOX-mediated activation of STAT5 in T-cells [11]. In addition, a decrease in oxidants by the antioxidant N-acetyl cysteine (NAC) leads to dephosphorylation and thus inactivates STAT5 in human-activated memory B-cells [12]. Moreover, in acute myeloblastic leukemia (AML), a decrease in H_2_O_2_ production due to the knock-down of the p22phox NOX subunit attenuated STAT5 phosphorylation and the expression of its target Pim-1 [13].

In cHL, the role of oxidants in the survival and proliferation of HRS cells, particularly in relation to the activity of the JAK/STAT signaling pathway, remains largely unclear. Here, we demonstrate that cHL cell lines exposed to antioxidants or NOX inhibitors showed increased apoptosis. This augmented apoptosis was accompanied by diminished JAK/STAT signaling. Consistently, an ectopic expression of a constitutively active STAT6 variant partially restores the survival of cHL cells. Based on this, we hypothesize that inhibition of JAK/STAT signaling upon oxidant reduction is responsible for increased cell death. Taken together, our results shed more light on the importance of oxidants in regulating crucial survival pathways in HRS cells of cHL and imply that modulation of oxidant production could be a therapeutic option.

## 2. Materials and Methods

### 2.1. Cell Culture and Transfection

The classical Hodgkin’s lymphoma cell lines KM-H2, L1236, L428, SUP-HD1, and SU-DHL4 were purchased from the Leibniz-Institut-German collection of microorganisms and cell lines (DSMZ, Braunschweig, Germany). The human lung adenocarcinoma cell line H1975 as well as the breast adenocarcinoma cell line MDA-MB-436 were obtained from the American Type Culture Collection (ATCC, Manassas, VA, USA). The cell lines U-HO1, LCL POF7, LCL POF14, LCL POF21, DLBCL1, DLBCL2, DLBCL3, Med-B1, and the L428 derivate L428ST6VT, ectopically expressing the constitutively active STAT6VT protein, were generated in our laboratory (Institute of Pathology, Ulm University, Germany). All cell lines were grown in suspension in Iscove/RPMI-1640 medium (4:1) supplemented with 10% fetal calf serum, L-glutamine (2 mM), penicillin (100 U/mL), and streptomycin (100 U/mL) in 5% CO_2_ in a humidified atmosphere at 37 °C (Thermo Fisher Scientific, Walthman, MA, USA). Transfection of L428 cells was performed using the Amaxa nucleofector kit V (Lonza, Basel, Switzerland), according to the manufacturer’s protocol. Stable cell lines were generated by selecting transfected clones with 2 mg/mL G418 sulfate and maintenance in a medium containing 0.5 mg/mL G418 sulfate. Transfection of L428 cells with siRNA was performed using the Amaxa nucleofector kit V. For each transfection, 1.5 × 10^6^ cells were mixed with 15–30 pmol of the appropriate siRNA before transfection.

### 2.2. Antibodies, Plasmids, and siRNAs

Following antibodies were used: anti-FLAG (F7425, Sigma Aldrich, Darmstadt, Germany), anti-STAT6 (M-200; sc-1698; Santa Cruz Biotechnology, Dallas, TX, USA), anti-pSTAT6 (Y-641; Cell Signaling Technology, Danvers, MA, USA), anti-β-actin (SP124, Sigma Aldrich, Darmstadt, Germany), anti-PTP1B (H-135, sc14021, Santa Cruz Biotechnology, Dallas, TX, USA), anti-TC-PTP (TC45, no. 15095, Cell Signaling Technology, Danvers, MA, USA), anti-pTyr (P-Tyr-100, Cell Signaling Technology, Danvers, MA, USA), anti-cleaved Caspase 3 (Asp175; Cell Signaling Technology, Danvers, MA, USA), and anti-ERK2 (C-14; sc-154; Santa Cruz Biotechnology, Dallas, TX, USA). The expression vector encoding the constitutive active STAT6VT mutant was a kind gift from Dr. Olga Ritz (Institute of Pathology, Ulm University, Germany). siRNAs specific for PTPN1/PTP1B and PTPN2/TC-PTP were purchased from Ambion (Austin, TX, USA) and Qiagen (Hilden, Germany).

### 2.3. Preparation of Cell Extracts

Whole cell extracts were prepared using lysis buffer (20 mM Tris pH 8.0, 200 mM NaCl, 1% Triton X-100, 1 mM Dithiothreitol (DTT), and protease and phosphatase inhibitor tablets (Roche, Mannheim, Germany). Cytoplasmic and nuclear extracts were prepared using buffer A (10 mM 4-(2-hydroxythyl)-1-piperazineethanesulfonic acid (HEPES) pH 7.9, 10 mM KCl, 0.1 mM Ethylenediaminetetraacetic acid (EDTA), 0.1 mM Ethylene glycol-bis-(β-aminoethyl ether)-N,N,N′,N′-tetraacetic acid (EGTA), 1 mM DTT, and 0.5 mM Phenylmethylsulfonyl fluoride (PMSF) and buffer C (20 mM HEPES pH 7.9, 0.4 M KCl, 1 mM EDTA, 1 mM EGTA, 1 mM DTT, 1 mM PMSF, and 1% NP-40) respectively or the NE-PER Nuclear and Cytoplasmic Extraction Reagents Kit from Thermo Fisher Scientific (Walthman, MA, USA). Protease inhibitors were added to both methods (Roche Applied Sciences, Mannheim, Germany).

### 2.4. Immunoblot Analysis

Initially, 10 to 30 µg of whole cell or nuclear/cytoplasmic protein extracts were subjected to separation by SDS-PAGE and transferred to a nitrocellulose blotting membrane (GE HealthCare Life Sciences and cytiva) using standard protocols. The membrane was blocked with 5% milk in TBS-Tween 20 (TBS-T) for 1 h at room temperature and then incubated overnight at 4 °C with primary antibodies at a dilution of 1:1000 in TBS-T. The membranes were washed with TBS-T thrice and incubated for one hour with the corresponding horseradish peroxide conjugated secondary antibody at a dilution of 1:5000 to 1:10,000. The proteins were detected using ECL substrates (Thermo Scientific, Walthman, MA, USA).

### 2.5. Electromobility Shift Assay (EMSA)

Between 5–10 μg of nuclear protein extract from the treated or control cells was incubated along with 32P-labeled STAT6 specific oligonucleotides (5′-AGGTCGACTTCCCAAGAACAGA-3′/5′-AGGCTCTGTTCTTGGGAAGTCGA-3′), 1–2 μg Poly[d(I-C)] and 2–3 μL of binding buffer. The protein extracts were separated on a 5% native polyacrylamide gel and subjected to electrophoresis. The gel was dried and exposed to X-ray films.

### 2.6. Intracellular Oxidant Measurement

Cells were treated either with 500 µM of the NOX-inhibitor apocynin (Acetovanillon, Sigma-Aldrich, Darmstadt, Germany), 300 µM of the antioxidant butylated hydroxyanisole (BHA) (Sigma-Aldrich, Darmstadt, Germany), 10 µM of the flavoenzyme inhibitor diphenyleneiodonium (DPI) (Sigma-Aldrich, Darmstadt, Germany), or dimethyl sulfoxide (DMSO)as solvent control for 24 h. Additional cells were treated with 1,2-bis(o-aminophenoxy)ethane-N,N,N′,N′-tetraacetic acid-(acetoxymethyl ester) (BAPTA-AM) (Hycultec, Beutelsbach, Germany) and N-acetylcysteine (Sigma Aldrich, Darmstadt, Germany) for 1 h or were treated for 30 min with 10 µM H_2_O_2_. The cells were centrifuged and resuspended in 100 µL phosphate-buffered saline (PBS) and subsequently incubated with 10 µM 2′,7′-dichlorodihydrofluorescein diacetate (CM-H2DCFDA) (Hycultec, Beutelsbach, Germany) for 20 min at 37 °C. Dead cells were marked by staining with 50 µg/mL propidium iodide (PI) for another 5 min at room temperature incubated in the dark. The samples were measured by flow cytometer (Gallios, Beckman Coulter, Brea, CA, USA), each for 10,000 events.

### 2.7. Mitochondrial Superoxide Measurement

Between 500,000 to 1 million cells were seeded in 6 well plates and treated for 24 h with Apo, BHA, DPI, or DMSO as solvent control. Two hours prior to staining, additional cells were treated with NAC for 2 h, or BAPTA-AM for 1h. Samples were centrifuged at 500 rpm for 5 min and resuspended in 100 µL PBS. Cells were stained with 1.87 µM MitoSOXTM Red (Thermo Fisher Scientific, Walthman, MA, USA) and 10 µM H2DCFDA for 30 min at 37 °C in the dark. MitoSOXTM Red is selectively oxidized by superoxide, but not by other ROS or reactive nitrogen species (RNS), and is used for the detection of mitochondrial superoxide in living cells. After staining, cells were washed twice with PBS, resuspended in PBS, and incubated with SYTOX Blue (Thermo Fisher Scientific, Walthman, MA, USA) for 1 min to discriminate dead from viable cells. Samples were measured on BD LSR Fortessa (BD Biosciences, San Jose, CA, USA), acquiring 15,000 events per sample. Gating strategy was based on unstained control and single stained samples (MitoSOX, H2DCFDA, SYTOX Blue).

### 2.8. RT-qPCR

The RNeasy RNA extraction kit (Qiagen) was used to extract RNA from the cell lines, and 1–2 µg of total RNA was used for the reverse transcription reaction using the Superscript™ II reverse transcriptase Kit (Invitrogen, Life Technologies, Carlsbad, CA, USA) according to the manufacturer’s protocol. The real-time PCR was performed using the iQ™ SYBR green supermix (Bio-Rad Laboratories, Hercules, CA, USA). Each PCR reaction and gene expression was analyzed relative to the expression of the housekeeping gene (β-actin). The primers for amplification of NOX3, NOX5, NOX1, SOD1, SOD2, and GPX1 were obtained from RealTimePrimers.com. For NOX2 amplification, we used primers with the following sequences: 5′-ACCGGGTTTATGATATTCCACCT-3′ and 5′-GATTTCGACAGACTGGCAAGA-3′). Different cell lines representing specific lymphoma subtypes were grouped as follows: cHL included L428, L1236, KM-H2, U-HO1, and SUP-HD1; DLBCL included DLBCL2, DLBCL3, DLBCL4, SU-DHL4, Med-B1, and U2940; LCL included LCL POF7, LCL POF14, and LCL POF21. Cell lines that did not express the target gene were excluded from the analysis.

### 2.9. Cell Proliferation and Viability Assay

The cell lines were treated with various concentrations of apocynin, BHA, DPI, or DMSO (control) for 48 h. Equal volume of cells were incubated with 1:4 dilution of alarma blue^®^ solution (AbD Serotec, Kidlington, UK) for 1–4 h. After incubation the fluorescence intensity (excitation = 560 nm, emission = 590 nm) was measured using SPECTRAMaxGeminiEM fluorometer to determine the difference in proliferation of the cell lines compared to the control.

### 2.10. Cell Count

Cells were seeded at a density of 0.6 × 10^6^ cell/mL at the start of the experiment and were treated for 24 h or a 48 h with apocynin, BHA, DPI, or DMSO as indicated. Subsequently, the cells were counted using a trypan blue exclusion assay on a Vi-Cell Beck-man Coulter. Percentage viability was calculated by normalizing to the corresponding DMSO controls.

### 2.11. Measurement of Apoptosis

Initially, 10^6^ cells were seeded into a 6-well plate and treated with 500 µM apocynin, 300 µM BHA, 25 µM DPI, and DMSO as solvent control for 24 h. The cells were stained with FITC Annexin V and Propidium Iodide Solution (PI) using the FITC Annexin V Apoptosis Detection Kit with PI (BioLegend, San Diego, CA, USA). The stained samples were measured by flow cytometer (Gallios, Beckman Coulter) for 500,000 events or 5 min.

### 2.12. Transcriptome Analyses

Cell lines L428 and U-HO1 were treated with either 250 µM apocynin, 200 µM BHA, or 25 µM DPI for 24 h. Subsequently, RNA isolation was performed using the RNeasy Kit (Qiagen, Hilden, Germany) by following the instructions of the manufacturer, and 10 ng of purified RNA was used with the QIAseq UPX 3′ Transcriptome Kit from Qiagen to create the libraries according to the manufacturer’s protocol. The quality of the RNA and libraries were checked on the TapeStation 4150 from Agilent using the Kits RNA Screen Tape Assay and High Sensitivity D5000 Screen Tape (Agilent Technologies, Santa Clara, CA, USA). Sequencing was done using the NextSeq 550 Sequencing System with the NextSeq 550 System High-Output Kit (Illumina, San Diego, CA, USA). Sequencing data were demultiplexed using a QIAGEN CLC Genomics Workbench and were further analyzed with R, GSEA (Gene Set Enrichment Analysis), and Qiagen Gene Globe. Publicly accessible codes and databases were used.

### 2.13. Statistical Analysis

All statistical tests were performed using the OriginPro (Origin 2022) or GraphPad Prism software (Prism 7.0c, Prism 10.x). Values were first examined for normal distribution using the Shapiro–Wilk test. If the data were normally distributed, the significance was calculated using one-way ANOVA. However, if the normal distribution was not reached, the significance of the values was checked using the Mann–Whitney test. Significance was inferred at *p* < 0.05 (* *p* ≤ 0.05; ** *p* ≤ 0.01; *** *p* ≤ 0.001; **** *p* ≤ 0.0001).

## 3. Results

### 3.1. Treatment with Apo, BHA, or DPI Impairs Growth and Induces Apoptosis of cHL Cell Lines

**Figure 1:**
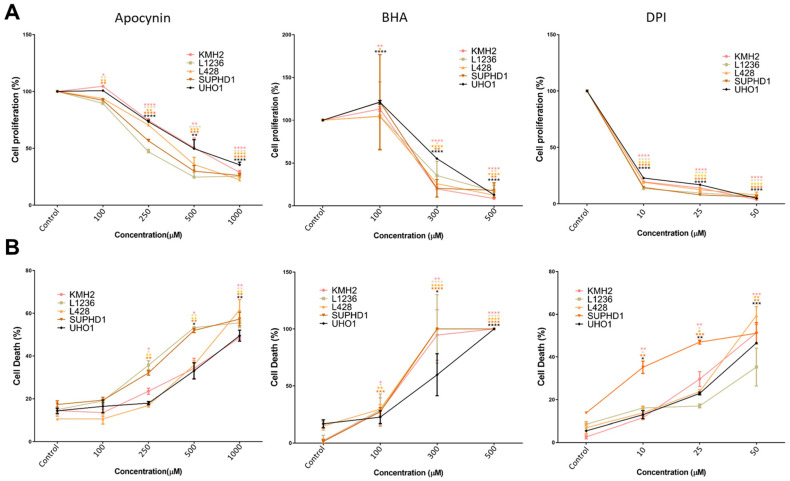
Treatment with Apo, BHA, or DPI causes attenuated cell growth and increased cell death of cHL cell lines. (**A**): Cell proliferation of the indicated cHL cell lines treated either with DMSO (Control) or with increasing concentrations of apocynin, BHA, or DPI for 48 h as measured by alamarBlue staining. The cell numbers obtained in the control samples were arbitrarily set to 100%, *n* = 3. (**B**): Cell death of the indicated cHL cell lines treated either with DMSO (Control) or with increasing concentrations of apocynin, BHA, or DPI for 48 h. Cells were stained with trypan blue and counted using ViCell Coulter, *n* = 3. * *p* < 0.05; ** *p* < 0.01; *** *p* < 0.001; **** *p* < 0.0001.

HRS cells and cHL cell lines are known to exert constitutively engaged IL4 and IL13 signaling [3,4], which has been shown to essentially involve NOX-mediated oxidants production in colon cancer [14]. To determine whether NOX-mediated oxidants production affects the survival and proliferation of cHL cells, we treated a panel of different cHL cell lines (L428, L1236, KMH2, U-HO1, and SUPHD1) with increasing concentrations of apocynin (Apo) and diphenyleneiodonium (DPI), or the antioxidant butylated hydroxyl anisole (BHA). We determined the impact on cell proliferation and cell death. Treatment of cells with Apo, BHA, or DPI caused a dramatic dose-dependent reduction in the cell numbers of all cHL cell lines tested (Figure 1A, Supplementary Figure S1). In addition, the percentage of cell death increased in a concentration-dependent manner, reaching a fraction of 50% in the case of 1 mM Apo or 50 µM DPI and nearly 100% using 300–500 µM BHA (Figure 1B). In Supplementary Figure S9, lower DPI concentrations (1–25 µM) were examined in two cHL cell lines, one DLBCL cell line (SU-DHL4), one PMBL cell line (Med-B1), one lung adenocarcinoma cell line (H1975), and one breast adenocarcinoma cell line (MDA-MD-436), revealing a decrease in cell viability with increasing DPI concentrations in all cell lines except MDA-MB-436, which showed only a ~7% reduction in viability at 25 µM DPI. A similar weak effect on viability was observed for Apo and BHA in Supplementary Figure S1, where only 500 µM BHA reduced viability by about 16%.

**Figure 2:**
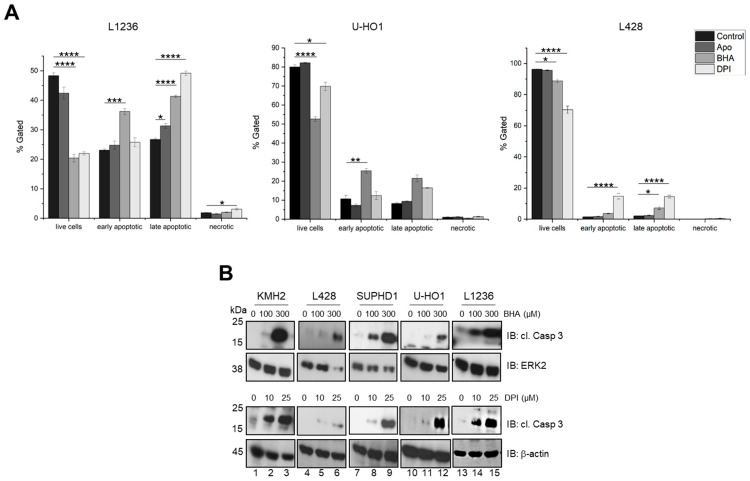
Apoptosis is induced in cHL cell lines by Apo, BHA, and DPI treatment. (**A**): Measurement of induced apoptosis in different cHL cell lines treated for 24 h either with DMSO as control, 500 µM apocynin, 300 µM BHA, or 25 µM DPI. Cells were stained with AnnexinV/PI and analyzed by FACS (*n* = 3). * *p* < 0.05; ** *p* < 0.01; *** *p* < 0.001; **** *p* < 0.0001. (**B**): Immunoblot analysis of whole cell extracts derived from cHL cell lines treated with increasing concentrations of BHA or DPI for 24 h using antibodies specific for either cleaved caspase-3 (cl. Casp 3), ERK2, or β-actin.

To unravel the molecular mechanism causing cell death after treatment of the cHL cell lines with Apo, BHA, or DPI, apoptotic cells were determined by annexin staining measured by FACS. In all three cell lines tested (L428, U-HO1, and L1236), we saw a significant increase in early and late apoptotic cells and a significant decrease in living cells (Figure 2A, Supplementary Figure S2). Similarly, treatment with either BHA or DPI caused a distinct increase in cleaved caspase-3 in all cHL cell lines analyzed, supporting the notion that NOX inhibition or antioxidant treatment induced apoptosis (Figure 2B).

### 3.2. Treatment with Apo, BHA, or DPI Reduces Cellular Oxidant Levels in cHL Cell Lines

**Figure 3:**
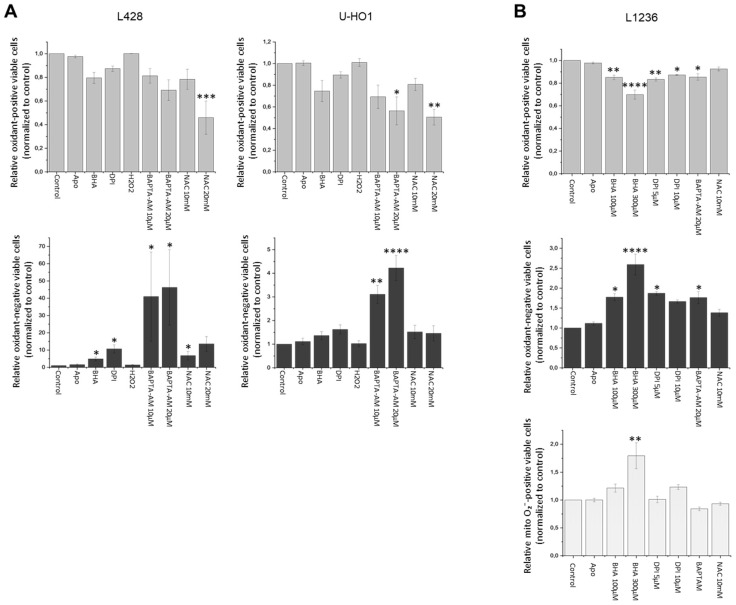
Apo, BHA, and DPI reduce cellular oxidant levels in cHL cell lines, but do not affect or increase mitochondrial superoxide. (**A**): Intracellular oxidants levels were measured in L428 and U-HO1 cell lines. Oxidant-positive viable cells (upper panel) as well as oxidant-negative viable cells (lower panel) normalized to control. (**B**): Oxidant-positive (upper panel) or -negative (middle panel) viable L1236 cells. The mitochondrial superoxide level was measured in L1236 cells (lower panel). All cells were treated with Apo (500 µM), 100 or 300 µM BHA, or 5 or 10 µM DPI for 24 h, as well as BAPTA-AM and NAC for 1 h, and H_2_O_2_ (10 µM) for 30 min. Superoxide-positive viable cells as well as superoxide-negative viable cells normalized to control. Oxidants levels were assessed using H2DCFDA/PI (**A**) or MitoSOX/SYTOXTM Blue (**B**) followed by flow cytometric analysis ((**A**) *n* = 4) ((**B**) *n* = 3). * *p* < 0.05; ** *p* < 0.01; *** *p* < 0.001; **** *p* < 0.0001.

To determine the efficacy of Apo, BHA, or DPI on intracellular oxidants, the cHL cell lines L428, U-HO1, and L1236 were used, and oxidant levels were quantified using CM-H2DCFDA. To evaluate the effect on cellular oxidant levels, three aspects were taken into account: 1. The number of viable oxidant-positive cells; 2. The number of viable oxidant-negative cells; and 3. The number of dead cells caused by the treatment. Application of BHA and DPI consistently reduced intracellular oxidants, as demonstrated by the reduced number of viable oxidant-positive and an increased number of viable oxidant-negative cells, although not all reductions reached statistical significance, whereas Apo showed only a modest effect (Figure 3, Supplementary Figures S3 and S4). Moreover, as expected, we observed an increase in dead cells (Supplementary Figure S3) after treatment with BHA and DPI. As oxidant production by NOX or by mitochondrial respiration is calcium-dependent [15] L428, U-HO1, and L1236 cells were treated with the cell-permeable calcium chelator BAPTA-AM, similarly resulting in a significant increase of oxidant-negative cells. N-Acetylcysteine (NAC), which was used as an additional positive control [16], scavenged oxidants in L428 and U-HO1 significantly in a dose-dependent manner. However, 30–50% of cells died after oxidant inhibition at 20 mM NAC. To exclude the mitochondria as source of the oxidants, mitochondrial superoxide levels in L1236 cells were measured by MitoSOX after treatment with different compounds (Figure 3B, lower panel). In contrast to the diminished overall oxidant measurements in BHA- or DPI-treated L1236 cells, the mitochondrial superoxide levels remained stable, except for a significant increase following treatment with BHA (300 µM).

### 3.3. Expression Levels of Oxidative Stress Genes in cHL, DLBCL and LCL Cell Lines, and cHL Tumor Samples

**Figure 4:**
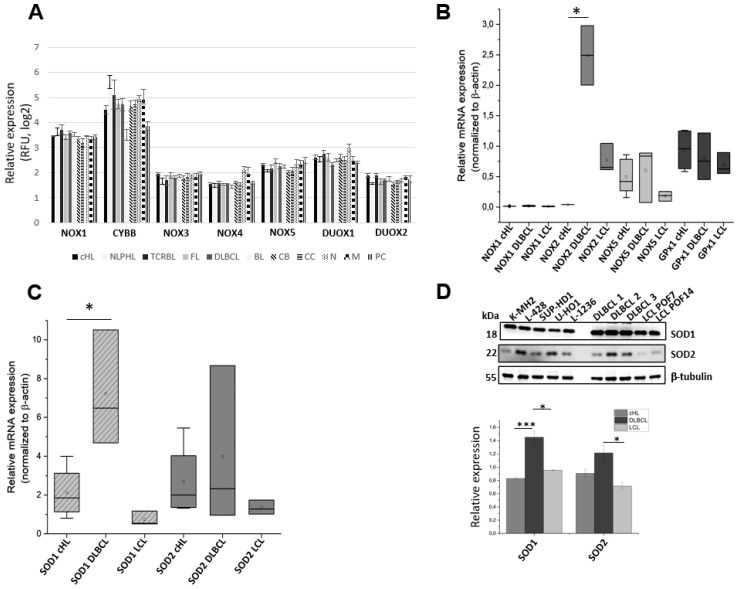
Analysis of oxidant-modifying factors in different lymphomas. (**A**): Relative expression levels of the indicated oxidant-modulating genes were compared across lymphoma entities, including nodular lymphocyte-predominant Hodgkin’s lymphoma (NLPHL), T-cell rich B-cell lymphoma (TCRBL), follicular lymphoma (FL), Burkitt’s lymphoma (BL), chronic B-cell lymphoma (CB), chronic lymphocytic leukemia (CLL), node (N), metastasis (M), and primary central nervous system lymphoma (PC). Re-analysis of GEP data set SE12453 (18794340) mined from the GEO database (www.ncbi.nlm.nih.gov/geo/) using the GeneSifter microarray data analysis software version 5.0 (www.genesifter.net, PerkinElmer, Waltham, MA), with RMA normalization applied; data are shown as mean fluorescence ± standard deviation (SD). (**B**): RT-qPCR analysis of NOX1, NOX2, NOX5, and GPx1 expressions were conducted in DLBCL (*n* = 2–3) and LCL (*n* = 3) cell lines, compared to cHL (*n* = 1–5). n indicates the number of cell lines within each group expressing the target gene. (**C**): RT-qPCR analysis of SOD1 and SOD2 expression was performed in DLBCL (*n* = 3) and LCL (*n* = 3) cell lines and compared to cHL cell lines (*n* = 4). n indicates the number of cell lines within each group expressing the target gene. (**D**): Protein expression of SOD1 and SOD2 was confirmed by immunoblotting in five cHL, three DLBCL, and two LCL cell lines, using β-tubulin as a housekeeper. * *p* < 0.05; *** *p* < 0.001.

Conflicting results were published regarding the ROS levels and the expression of the different NADPH oxidase (NOX) isoforms and subunits in HRS cells or cHL cell lines. While Gierfing et al. reported a reduced expression of components of the NOX complex in HRS and cHL cell lines accompanied by a decreased production of superoxide anion, Bur et al. demonstrated an increased expression of oxidative stress markers in aggressive Hodgkin’s lymphomas [17,18]. To determine the expression profile of crucial components of the cellular redox system, the gene expression profiling (GEP) data set SE12453 [19] mined from the GEO database (www.ncbi.nlm.nih.gov/geo, accessed on 28 November 2024) using the GeneSifter microarray data analysis software version 5.0 (www.genesifter.net, PerkinElmer, Waltham, MA) was reanalyzed. The analyses of several NOX family members, as well as *DUOX1* and *DUOX2*, across a panel of tumor samples showed no significant differences between cHL and other lymphoma subtypes (Figure 4A). The scale in panel 4A represents the relative expression of the indicated oxidase within the respective B-cell lymphoma samples and therefore allows comparison of the expression levels both between different B-cell lymphoma entities and among the individual oxidases. The expression levels of *NOX*, *SOD*, and *DUOX* genes were analyzed at the mRNA level. qPCR analysis revealed a significantly increased *NOX2* (*CYBB*) expression in DLBCL cell lines compared to LCL or cHL cells (Figure 4B). In contrast, *GPx1*, *NOX1*, and *NOX5* expression did not differ between these groups, and *NOX3* and *NOX4* expression were not detectable. Antioxidant enzymes *SOD1* and *SOD2* were also investigated regarding their mRNA and protein expression in cHL, LCL, and DLBCL cell lines. *SOD1* demonstrated significantly higher mRNA expression in DLBCL compared to cHL, while *SOD2* levels did not show significant differences between the groups (Figure 4C). On protein level, *SOD1* and *SOD2* are expressed at similar levels in cHL and LCL cells (Figure 4D). However, among the cHL cell lines, L428 and U-HO1 show the strongest SOD2 expression. The examined DLBCL cell lines exhibit increased expression of both SOD1 and SOD2 compared with cHL and LCL cells. Taken together, microarray analysis of cHL tumor samples did not reveal major differences in *NOX* or *DUOX* expression compared to other lymphoma entities (Figure 4A), whereas qPCR and immunoblot analysis showed higher NOX2 and SOD expression in DLBCL cell lines relative to cHL and LCL cell lines (Figure 4B–D).

### 3.4. Treatment with Apo, BHA, and DPI Affects Different Genes and Signaling Pathways

To investigate the impact of modulating the oxidant levels on gene expression in cHL, L428 and U-HO1 cells were treated with Apo, BHA, or DPI followed by transcriptome profiling. This revealed that Apo treatment had the mildest effect, as expected, while BHA and DPI treatment altered the expression patterns of both cell lines markedly (Supplementary Figures S5 and S6). Distinct gene clusters were specifically upregulated in Apo-, BHA-, or DPI-treated cells while being downregulated under other conditions (Supplementary Figure S5). Among DPI-treated U-HO1 cells, >85% of significantly altered genes were downregulated (e.g., FYB2, TTLL7, and ZPLD1). Few genes were upregulated (e.g., CCDC28B, HMOX1, IRF1, and TP53). BHA induced a more balanced expression shift, including upregulation of HACD4, TSC1, and multiple SNAR-associated genes, alongside downregulation of RAP1A, PIK3R1, and MAP7. In L428, DPI caused both up- (e.g., DPH2, TNFSF15, and MMP13) and downregulation (e.g., multiple SNAR-associated genes and COL19A1). BHA notably upregulated immune/inflammatory genes (e.g., MMP13 and TNFSF15) and downregulated various small nucleolar RNAs (SNARs). A Venn diagram of DPI- and BHA-treated U-HO1 and L428 cells revealed limited overlap in differentially expressed genes (Supplementary Figure S6). Only a few genes—LINC01203, MAPK9, RAP1A, TAOK1, and ZPLD1—were consistently affected across treatments and cell lines. These genes are linked to stress responses, cytoskeletal regulation, and cellular microenvironment interactions.

**Figure 5:**
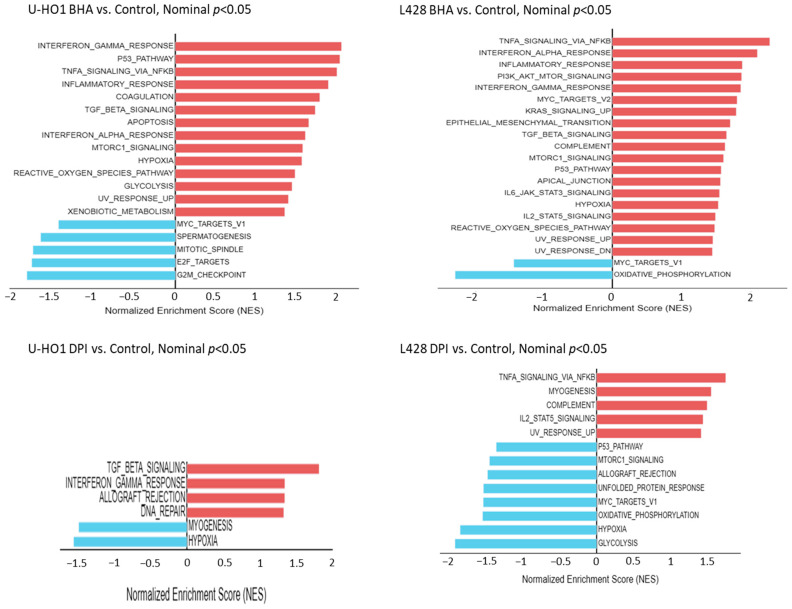
Transcriptome analysis in L428 and U-HO1 cells. Transcriptome profiling was performed to analyze hallmark gene expression in BHA- (200 µM), DPI- (25 µM), or DMSO-treated L428 and U-HO1 cells after 24 h of treatment compared to untreated controls. Gene set enrichment analysis (GSEA) was performed using hallmark gene sets from MSigDB. Shown are all hallmark pathways with *p* < 0.05, ranked by normalized enrichment score (NES).

An additional GSEA identified oxidant-responsive signaling pathways and cell line- or treatment-specific effects (Figure 5). BHA strongly induced stress response pathways in both cell lines (e.g., TNFα/NF-κB, interferon, ROS, p53, UV response, and mTORC1), while downregulating MYC_TARGETS_V1. DPI had milder effects in U-HO1, notably up-regulating TGF_BETA_SIGNALING and INTERFERON_GAMMA_RESPONSE, but triggered broader changes in L428, including downregulation of MYC_TARGETS_V1 and OXIDATIVE_PHOSPHORYLATION. Together, these GSEA results reveal highly heterogenous, compound, and cell line responses affecting different apoptotic and proliferative pathways, including Myc targets.

### 3.5. Apo, BHA, and DPI Affect Constitutively Active JAK/STAT Signaling in cHL Cell Lines

**Figure 6:**
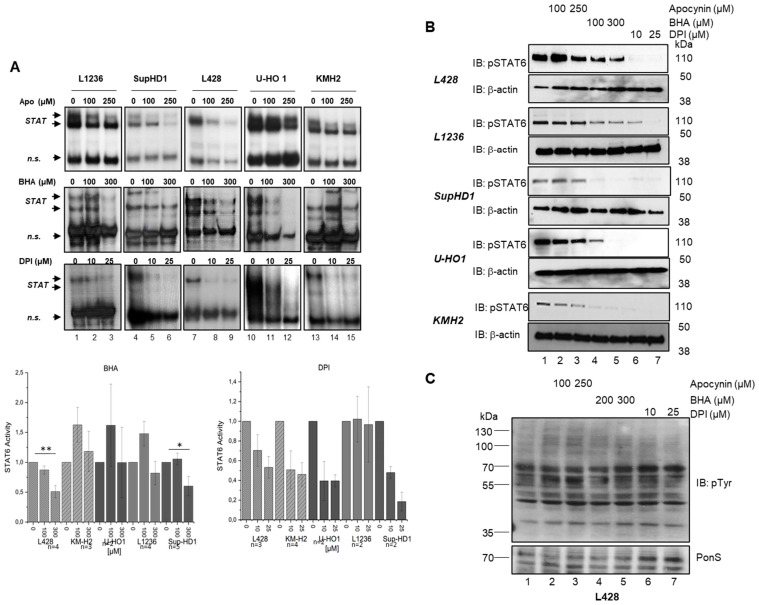
Treatment with Apo, BHA, and DPI attenuates JAK/STAT signaling in cHL cell lines. (**A**): Electrophoretic mobility shift assay (EMSA) using a 32P-labeled STAT-specific oligonucleotide and nuclear extracts from the indicated cHL cell lines treated with DMSO (0) or increasing concentrations of Apo, BHA, or DPI. * *p* < 0.05; ** *p* < 0.01 (**B**): Immunoblot analysis of whole cell extracts from the indicated cHL cell lines using antibodies specific for pSTAT6 and a β-actin as loading control. Cells were treated with DMSO, Apo, BHA, or DPI, as indicated. (**C**): Immunoblot analysis of whole cell extracts from L428 cells using an anti-phosphotyrosine (pTyr) antibody. Equal protein loading was verified by PonceauS (PonS) staining of the membrane. L428 cells were treated with either DMSO, Apo, BHA, or DPI for 24 h.

As we observed a devastating effect by inhibiting NOX-dependent oxidants’ production on the proliferation and survival of cHL cell lines, we next aimed to determine the molecular mechanism underlying this effect. Since the regulation of JAK/STAT pathways has been described previously as redox-sensitive [7,10], we analyzed the impact of Apo, BHA, and DPI treatment on the constitutive JAK/STAT signaling in cHL cell lines. Nuclear proteins isolated from the different cHL cell lines that were either treated with DMSO or with Apo (100 µM and 250 µM), BHA (100 µM and 300 µM), or DPI (10 µM and 25 µM) were subjected to electrophoretic mobility shift assay (EMSA) using a STAT specific radio-actively labeled oligonucleotide. In general, Apo, BHA, or DPI treatment caused a diminished STAT DNA binding activity in all cHL cell lines tested (Figure 6A). Moreover, western blot analysis of whole cell extracts from cHL cell lines treated with Apo, BHA, and DPI also revealed a reduction in STAT6 phosphorylation (Figure 6B). However, the extent of the phospho-STAT6 reduction varied along the different agents used. DPI consistently had the strongest impact, while BHA treatment led to a moderate decrease, and apocynin had only a minor effect on the phospho-STAT6 levels. The effect of Apo, BHA, and DPI treatment is not limited to phospho-STAT6 levels, but also a decrease in phospho-STAT3 levels is observed in L428 (Supplementary Figure S7A). Interestingly, however, U-HO1 cells showed no significant changes in phospho-STAT3 levels (Supplementary Figure S7B). To exclude whether treatment of the cHL cell lines had a general impact on intracellular tyrosine phosphorylation, we determined phospho-tyrosine levels by western blot analysis. As shown in Figure 6C and Supplementary Figure S8, total phospho-tyrosine levels remained unaltered upon treatment with Apo, BHA, or DPI, suggesting that the observed reduction in pSTAT6 levels is not due to a general reduction in tyrosine-phosphorylation.

### 3.6. Ectopic Expression of a Constitutively Active STAT6 Rescues Apo, DPI, or Antioxidant-Induced Cell Death

**Figure 7:**
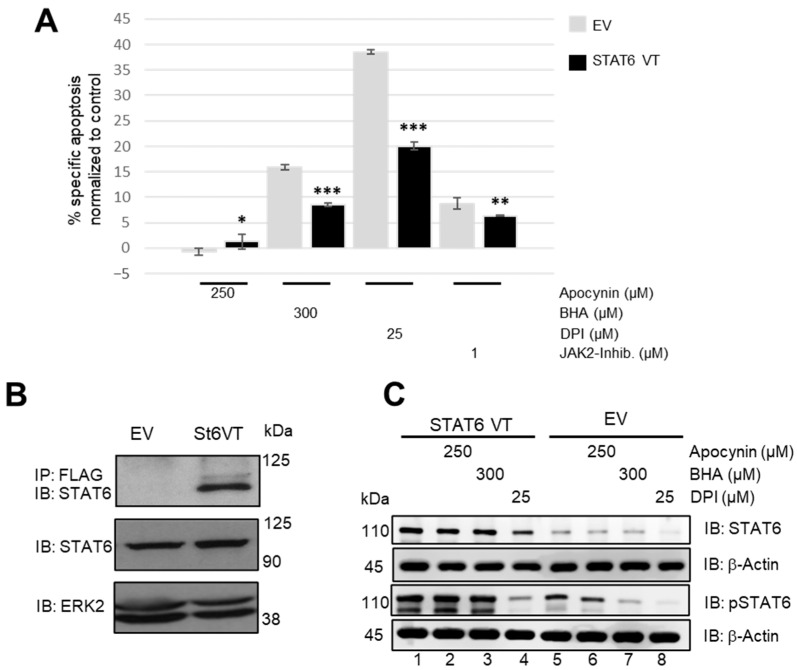
Ectopic expression of a constitutively active STAT6 mutant reduces cell death induced by Apo, BHA, or DPI treatment. (**A**): Apoptosis of the L428 control cells (empty vector, EV) and L428-STAT6VT cells were assessed after treatment with apocynin (250 μM), BHA (300 μM), DPI (25 μM), or a JAK2 inhibitor (1 µM) for 24 h. Apoptotic cells were quantified by Annexin V/PI staining followed by flow cytometric analysis *(n* = 3). * *p* < 0.05; ** *p* < 0.01; *** *p* < 0.001. (**B**): Characterization of the L428 cell clone stably expressing STAT6VT. Anti-FLAG immunoprecipitation was performed using whole cell extracts from EV and STAT6VT-expressing cells, followed by immunoblotting with a STAT6-specific antibody. Additionally, whole cell extracts were analyzed directly by immunoblot using antibodies against STAT6 and ERK2. (**C**): Immunoblot analysis of whole cell lysates from L428-EV and L428-ST6VT cells using antibodies specific for pSTAT6, total STAT6, and β-actin.

Our results suggest that inhibition of the JAK/STAT pathway might by responsible for the reduced proliferation and increased apoptosis in Apo-, BHA-, or DPI-treated cHL cell lines. To further underscore this finding, an L428 cell line stably expressing a constitutively active STAT6 mutant (STAT6VT) was generated (Figure 7B). STAT6VT cells showed a partial rescue from apoptosis induced by BHA (8% vs. 16% apoptotic cells) or DPI (20% vs. 39% apoptotic cells) treatment (Figure 7A). Moreover, STAT6VT expression also rescued L428 cells for apoptosis induced by treatment with a pharmacological JAK2 inhibitor, although to a lower extent (6% vs. 9% apoptotic cells). STAT6 phosphorylation was similarly affected by Apo, BHA, or DPI treatment in control L428 and STAT6VT-L428 cells (Figure 7C). However, basal STAT6 phosphorylation levels appeared to be higher in STAT6VT-L428 cells. Taken together, as STAT6VT activity is independent of JAK2 activity, the partial rescue of STAT6VT-L428 cells in combination with the similar decrease in pSTAT6 levels suggest that the apoptosis-inducing effect of Apo, BHA, or DPI treatment is partially based on an impairment of JAK2 activity.

### 3.7. Knockdown of Protein Tyrosine Phosphatases Attenuates Apo, BHA, or DPI-Induced Cell Death

**Figure 8:**
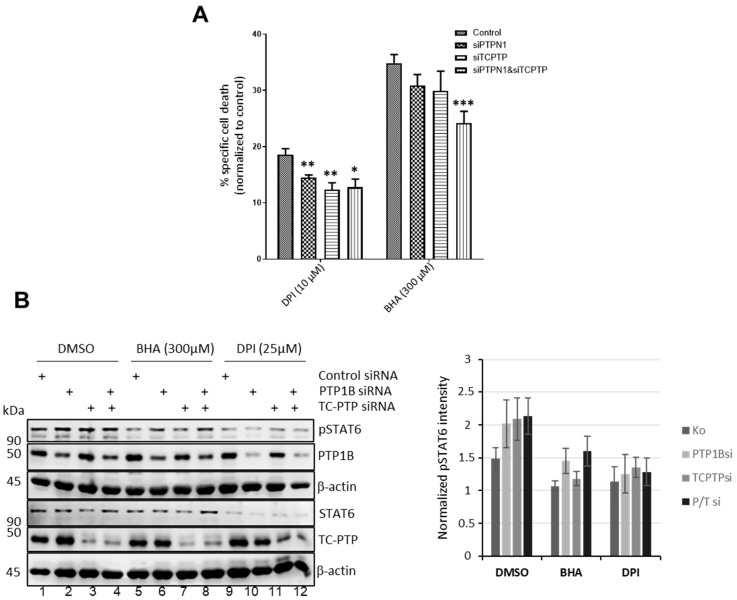
Knockdown of PTP1B or TC-PTP diminished BHA- and DPI-induced cell death in L428 cells. (**A**): Apoptosis in L428 cells transiently transfected with the indicated siRNAs was determined by Annexin V/PI staining followed by flow cytometric analysis. Cells were treated for 24 h with DMSO (Control), BHA (300 μM), or DPI (10 μM). Shown is the ratio of apoptotic cells normalized to DMSO-treated controls (*n* = 3). * *p* < 0.05; ** *p* < 0.01; *** *p* < 0.001. (**B**): Immunoblot analysis of transiently transfected L428 cells with indicated siRNAs following treatment with DMSO, BHA (300 µM), or DPI (10 µM). Antibodies used for detection include pSTAT6, STAT6, TC-PTP, PTP1B, and β-actin (*n* = 4).

As the JAK/STAT pathway appears to participate in the Apo-, BHA-, or DPI-induced effects, we next asked whether the redox-regulated protein tyrosine phosphatases PTP1B and TC-PTP [20], known to be negative regulators of the JAK/STAT signaling pathway, are involved in this redox-regulated process. To examine the potential role of PTP1B and TC-PTP, L428 cells were transiently transfected with siRNAs specific to PTP1B and TC-PTP. Determination of the percentage of specific cell death caused by either BHA or DPI treatment revealed reduced apoptosis in cells with either individual PTP1B or TC-PTP knockdown or the combined knockdown of PTP1B and TC-PTP (Figure 8A). In the case of BHA treatment, a combinatorial effect was observed when the expression of both PTPs was suppressed (Figure 8A). The effect of the PTP knockdown was also visible on phospho-STAT6 levels in untreated or BHA- and DPI-treated L428 cells. As expected, phospho-STAT6 levels were diminished after BHA or DPI treatment (Figure 8B). PTP1B knockdown, but not TC-PTP knockdown, partially reverted this BHA and DPI-induced reduction in STAT6 phosphorylation (Figure 8B), although not reaching statistical significance. Combined, the data suggest that PTP1B and TC-PTP are involved in the oxidant-dependent regulation of phospho-STAT6 levels in cHL cell lines.

## 4. Discussion

Oxidants have been linked to antimicrobial responses or defective respiratory chain activity, leading to undesirable modifications of cellular macromolecules like DNA or protein alterations [21,22,23]. However, accumulating evidence supports the notion that oxidants exert essential roles in various signal transduction processes [9,10]. NADPH-dependent oxidases (NOXs) are recruited to the plasma membrane upon stimulation by cytokines IL4 and IL13, resulting in temporally and spatially restricted H_2_O_2_ production. For instance, NOX-mediated oxidant generation has been shown to be essential for an IL4-induced activation of the JAK/STAT signaling pathway [4,15], which is also crucial for the survival and growth of HRS cells and cHL cell lines [4]. This link prompted us to explore the effect of NOX inhibition or oxidant scavenging on JAK/STAT activation in cHL cell lines. We focused on STAT6 because HRS cells exhibit constitutive activation of this transcription factor, which is involved in IL4/IL13 signaling, promoting cell survival and immune evasion [24]. We observed a distinct reduction in STAT6 DNA-binding activity as well as decreased phosphorylation of STAT6 at the tyrosine residue across all cHL cell lines used, pointing to a diminished STAT activity as basis of the negative effect imposed by Apo, BHA, or DPI. This result is consistent with the apocynin- or DPI-mediated impairment of STAT6 activity in A549 cells described by Sharma et al. [15]. Consistently, L428 cells with an ectopic expression of a constitutive active STAT6 (STAT6VT) show a reduced apoptosis (Figure 7A). The activity of the STAT6VT mutant is independent of the upstream JAK2 kinase, and the partial rescue of STAT6VT expressing L428 cells implies that inhibition of JAK2 is at least involved in the BHA and DPI effect on cHL cell line survival. However, the negative effect is not limited to STAT6 as also phospho-STAT3 levels decreased in L428 after treatment, which is in line with previous studies showing STAT3 is frequently activated in cHL and its microenvironment. However, STAT3 is considered a common but not defined feature of cHL pathogenesis [24].

Taking the crucial role of STAT activity for cHL proliferation and survival into account, the growth inhibition and reduced survival of cHL cell lines upon Apo, BHA, or DPI treatment are in line with these findings (Figure 1, Supplementary Figure S1). Similar results were obtained in a study using the EBV-positive Burkitt’s lymphoma cell line Raji, where the viral protein LMP1 increases H_2_O_2_ levels, and DPI-mediated blockade of H_2_O_2_ production induces cell cycle arrest and subsequent apoptosis [8,25]. In our experiments, we obtained evidence that the observed increase in cell death is caused by apoptosis, as demonstrated by FACS analysis and the appearance of cleaved caspase-3 (Figure 2, Supplementary Figure S2).

Based on our oxidant measurements, intracellular oxidant levels were found to decrease upon treatment with BHA or DPI (Figure 3 and Supplementary Figure S3), indicating that the observed cell death is associated with reduced oxidant levels affecting JAK/STAT signaling. This increase in apoptosis might also explain the relatively mild effect of Apo, BHA, or DPI treatment on intracellular oxidant levels, as apoptotic cells were excluded from the analysis (Figure 3). Furthermore, treatment with the calcium chelator BAPTA-AM also lowered intracellular oxidant levels, which is consistent with previously published data regarding the role of calcium in oxidant-mediated JAK/STAT activation in IL4-stimulated B cells [15]. However, it is noteworthy that calcium has versatile effects on cellular signaling. For instance, calcium might affect JAK/STAT signaling by a direct effect on NOX activity by modulating STAT phosphorylation mediated by CAMKII [26,27] or by affecting the stability of the negative JAK/STAT regulator protein phosphatase 1B [28]. Defining the exact points at which calcium affects JAK/STAT activity in cHL cell lines or HRS cells will be an important part of future studies.

Importantly, these effects were not due to compound-induced disruption of the mitochondrial respiratory chain. None of the compounds and concentrations used diminished mitochondrial superoxide production, a BHA treatment with 300 µM even increased mitochondrial superoxide levels. Notably, 300 µM BHA was the most effective condition in reducing intracellular oxidant levels in L1236 cells. Together, these findings support the notion that intracellular oxidants produced by NOX are critical for cHL cell survival, as treatment with Apo, BHA, or DPI did not reduce mitochondrial superoxide levels.

Cellular targets of the NOX-derived oxidants might be the non-receptor protein tyrosine phosphatases PTP1B and TC-PTP, which are known to be redox-sensitively regulated by temporary and reversible oxidation of a cysteine residue in their active sites [29,30,31,32,33]. Reduction of cellular PTP1B and/or TC-PTP levels by siRNA indeed reduced apoptosis in DPI or BHA-treated L428 cells (Figure 8A) accompanied by a less pronounced loss in STAT6 phosphorylation (Figure 8B). However, PTP1B and TC-PTP appear not to be the only targets as siRNA-mediated knock down of these phosphatases only partially restored L428 survival and STAT6 phosphorylation (Figure 8), although an incomplete knock down of both phosphatases might have hampered this analysis. This notion is supported by the observation that growth and survival of the PTP1B defective cHL cell line U-HO1 [34] is also affected by Apo, DPI, or BHA treatment (Figure 1) although less pronounced as compared to the other cHL cell lines used (Figure 1 and Supplementary Figure S1). The reduced response of U-HO1 to Apo, BHA and DPI is also highlighted by the stable phospho-STAT3 levels (Supplementary Figure S7B). Together, we concluded that PTP1B and TC-PTP participate in the oxidant-mediated regulation of the essential STAT activity, but are not the exclusive targets.

To further elaborate the connection between impairment of oxidant production, JAK/STAT activity and cHL, cell proliferation and survival, we employed transcriptomic analyses. Indeed, treatment of cHL cells with BHA and DPI induces consistent transcriptomic alterations characterized by the suppression of proliferative gene programs (MYC, E2F, and cell cycle regulation) and metabolic pathways (glycolysis and oxidative phosphorylation), alongside activation of immune and stress responses including interferon signaling, TNFα/NFκB, inflammation, apoptosis, and hypoxia. These findings are consistent with previous reports highlighting the role of redox homeostasis in regulating essential cellular processes. BHA is known to stimulate the HO-1/Nrf2 axis and induce p53, interferon-, and NFκB-signaling pathways, promoting cell cycle arrest [35,36,37,38]. DPI exhibits a differential effect on p53-dependent and -independent cell cycle progression, inhibiting proliferative and metabolic signaling while enhancing immunological and apoptotic responses [37]. Despite impaired STAT6 and STAT3 activation following BHA and DPI treatment, we observe strong induction of inflammatory and interferon-mediated gene sets. This paradox may be explained by apoptotic cell death-induced release of damage-associated molecular patterns (DAMPs), which can amplify secondary immune signaling, probably by NFκB-signaling. In line with this, DPI induces G2-M cell cycle arrest in Raji cells, leading to apoptosis, which was accompanied by c-Myc downregulation and a reduction in oxidant levels [8]. In aggressive NK-cell leukemia (ANKL), STAT3 signaling was shown to act as an upstream regulator that directly activates the MYC transcriptional program [39]. Overall, our transcriptome data indicate that modulation of ROS levels by BHA or DPI in cHL cells leads to coordinated suppression of proliferation and metabolism alongside activation of immune and apoptotic pathways. These effects, occurring despite impaired STAT3/6 activation, highlight the therapeutic potential of targeting redox homeostasis in cHL lymphoma.

Analyzing the expression levels of the different NAPDH-dependent oxidases, we observed low mRNA levels of *NOX1* and *NOX2* and normal levels of *NOX5* in the cHL cell lines (Figure 4B). In cHL tumor samples, however, no significant differences in the expression of a panel of redox regulators were observed compared to other types of B cell lymphoma (Figure 4A). These findings are in line with the results presented by Giefing et al., who reported downregulation of *NOX2/CYBB* and other genes in cHL cell lines compared to normal B cells, resulting in impaired NADPH oxidase function and reduced oxidant production in cHL [18]. Oxidant production might differ between cHL tumor samples and cHL cell lines due to differences in *NOX* gene expression, and we also observed variability in *NOX* expression among the cHL cell lines themselves. However, as NOX inhibition and antioxidant scavenging by BHA shows a deleterious effect on cHL proliferation and survival, accompanied by an inhibition of STAT activity, we conclude that even a comparable low NOX-mediated oxidant production is required for cHL cell stability.

**Figure 9:**
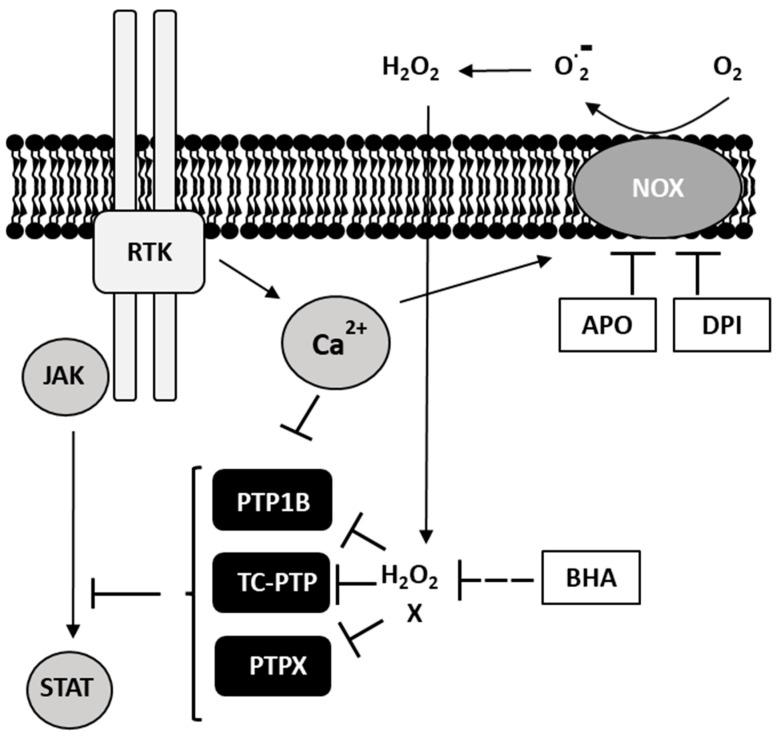
Schematic model illustrates the proposed oxidant-dependent signaling pathway in cHL cell lines and HRS cells. Oxidants generated by NOX, whose activity can be further supported by Ca^2+^-dependent mechanisms, promote activation of the JAK/STAT signaling pathway by inhibiting the negative regulatory phosphatases PTP1B and TC-PTP and potentially additional PTPs (PTPX). Pharmacological treatment with Apo, BHA, and DPI lowers intracellular oxidants levels, thereby relieving the inhibition of these phosphatases. This, in turn, leads to an overall reduction in JAK/STAT pathway activity. X indicates yet-unknown mechanisms that may also affect PTP activity upon treatment with Apo, BHA, and DPI.

Taken together, we would like to postulate the following model in Figure 9 for the role of the NOX-produced oxidants in the cell signaling of cHL cell lines and probably for the Hodgkin–Reed/Sternberg cells in classical Hodgkin’s lymphoma: Elevated cytokine levels in the cHL microenvironment lead to a permanent engagement of cytokine receptors, including IL4/IL13 receptors, causing constitutive activation of Janus kinases like JAK2 [3,4]. Activated JAKs phosphorylate and therefore activate STAT transcription factors, controlling anti-apoptotic and growth-supporting gene expression. In addition, ligand-bound cytokine receptors RTKs can trigger Ca^2+^ signals that further support NOX activation and local superoxide anion formation. This superoxide is converted to hydrogen peroxide by SODs. Hydrogen peroxide oxidizes specific proteins, including PTP1B and TC-PTP, which are subsequently inhibited. In addition, the activity of these phosphatases is likely modulated by further, yet-unknown mechanisms (indicated as X). As PTP1B and TC-PTP are known as negative regulators of the JAK/STAT pathway in lymphoma, inhibition of these phosphatases supports the growth and survival of HRS cells and cHL cell lines. Consistently, the inhibition of NOX-mediated oxidant generation by Apo, BHA, or DPI treatment reduces hydrogen peroxide levels and reactivates PTP1B and TC-PTP, which in turn dephosphorylate JAK2 and STAT6, causing a reduced proliferation and cell survival. As we observed a similar negative effect of DPI treatment on the likewise JAK/STAT-dependent cell lines SU-DHL4, Med-B1, and H1975 [3,4,40,41], but not on the JAK/STAT-independent cell line MDA-MB-436 [42] in a viability assay, we hypothesize that a decrease of NOX-derived cellular oxidants might be a general mechanism to impair JAK/STAT-dependent tumor cells, similar to the model of Konaté et al. As HRS cells, the malignant cell type in classical Hodgkin’s lymphoma, require and actively generate a specific cytokine and chemokine milieu for their survival, a constant production of oxygen species is an essential part of this process. Our results underscore that this constitutive oxidant production is essential for the survival of cHL cell lines. Disruption of this production leads to the induction of apoptotic processes in cHL cells and is associated with downregulation of JAK/STAT signaling.

## 5. Conclusions

In this study, we demonstrate that NOX-derived oxidants play a critical role in maintaining survival and proliferative signaling in classical Hodgkin lymphoma cell lines. Pharmacological inhibition of NOX activity or scavenging of intracellular oxidants results in reduced intracellular oxidant levels, attenuation of constitutive JAK/STAT signaling and induction of apoptosis. These effects are accompanied by transcriptional reprogramming characterized by suppression of proliferative and metabolic pathways and activation of stress, immune, and apoptotic responses. Mechanistically, our data indicate that redox-sensitive protein tyrosine phosphatases, including PTP1B and TC-PTP, contribute to oxidant-mediated regulation of STAT activity, although additional targets are likely involved. Importantly, the observed effects are independent of mitochondrial superoxide production, underscoring the specific relevance of NOX-derived oxidants. Together, our findings support a model in which a finely tuned redox balance is essential for cHL cell survival and suggest that targeting redox homeostasis may represent a promising strategy to impair JAK/STAT-dependent tumor growth in cHL.

## Data Availability

The original contributions presented in this study are included in the article/Supplementary Materials. Further inquiries can be directed to the corresponding author.

## Supplementary Materials

The following supporting information can be downloaded at: https://www.mdpi.com/article/10.3390/antiox15010090/s1, Figure S1: Treatment with Apo, BHA, or DPI affects cell viability; Figure S2: Quantification of cell death reveals distinct populations of live, early apoptotic, late apoptotic, and necrotic cells in cHL cell lines following Apo, BHA, or DPI treatment; Figure S3: Determination of ROS levels and cell viability in cHL cell lines following Apo, BHA, or DPI treatment; Figure S4: Quantification of intracellular oxidants levels in cHL cell lines following Apo, BHA, or DPI treatment; Figure S5: Unsupervised clustering of differentially expressed genes in L428 and U-HO1 cells after Apo, BHA, or DPI treatment; Figure S6: BHA and DPI treatment induce significant gene expression in L428 and U-HO1 cells; Figure S7: Treatment with Apo, BHA, and DPI attenuates pSTAT3 signaling in L428 and U-HO1; Figure S8: Global tyrosine phosphorylation (pTyr) profiles in cHL cell lines are not altered by Apo, BHA, or DPI treatment; Figure S9: DPI treatment affects cell viability differently across cell types.

## Abbreviations

The following abbreviations are used in this manuscript:

DLBCL	Diffused large B cell lymphoma
DMSO	Dimethyl sulfoxide
NOX	NADPH oxidase
JAK	Janus kinase
STAT	Signal Transducers and Activators of Transcription
LCL	Lymphoblastoid cell line
pRB	Phosphor-Retinoblastoma
pTyr	Phosphor-tyrosine
H_2_O_2_	Hydrogen peroxide
O_2_^−^	Superoxide
HRS	Hodgkin and Reed/Sternberg
Apo	Apocynin
BHA	Butylated hydroxyanisole
DPI	Diphenyleneiodonium chloride
cHL	Classical Hodgkin’s Lymphoma
IL	Interleukin
SOD	Superoxide dismutase
NAC	N-acetylcysteine
GSEA	Gene Set Enrichment Analysis
PTP1B	Protein-tyrosine phosphatase 1B
TC-PTP	T-cell protein tyrosine phosphatase

## References

[1] Küppers R. (2009). The biology of Hodgkin’s lymphoma. Nat. Rev. Cancer.

[2] Richardson S.E., McNamara C. (2011). The Management of Classical Hodgkin’s Lymphoma: Past, Present, and Future. Adv Hematol..

[3] Steidl C., Connors J.M., Gascoyne R.D. (2011). Molecular pathogenesis of Hodgkin’s lymphoma: Increasing evidence of the importance of the microenvironment. J. Clin. Oncol..

[4] Skinnider B.F., Mak T.W. (2002). The role of cytokines in classical Hodgkin lymphoma. Blood.

[5] Schindler C., Levy D.E., Decker T. (2007). JAK-STAT signaling: From interferons to cytokines. J. Biol. Chem..

[6] Karpathiou G., Papoudou-Bai A., Ferrand E., Dumollard J.M., Peoc’h M. (2021). STAT6: A review of a signaling pathway implicated in various diseases with a special emphasis in its usefulness in pathology. Pathol. Res. Pract. Volume.

[7] Liou G.Y., Storz P. (2010). Reactive oxygen species in cancer. Free. Radic. Res..

[8] Ding Y., Zhu W., Sun R., Yuan G., Zhang D., Fan Y., Sun J. (2015). Diphenylene iodonium interferes with cell cycle progression and induces apoptosis by modulating NAD(P)H oxidase/ROS/cell cycle regulatory pathways in Burkitt’s lymphoma cells. Oncol. Rep..

[9] Spencer N.Y., Engelhardt J.F. (2014). The basic biology of redoxosomes in cytokine-mediated signal transduction and implications for disease-specific therapies. Biochemistry.

[10] Storz P. (2005). Reactive oxygen species in tumor progression. Front. Biosci..

[11] Shatynski K.E., Chen H., Kwon J., Williams M.S. (2012). Decreased STAT5 phosphorylation and GATA-3 expression in NOX2-deficient T cells: Role in T helper development. Eur. J. Immunol..

[12] Bonnaure G., Néron S. (2014). N-acetyl cysteine regulates the phosphorylation of JAK proteins following CD40-activation of human memory B cells. Mol. Immunol..

[13] Woolley J.F., Naughton R., Stanicka J., Gough D.R., Bhatt L., Dickinson B.C., Chang C.J., Cotter T.G. (2012). H2O2 production down-stream of FLT3 is mediated by p22phox in the endoplasmic reticulum and is required for STAT5 signalling. PLoS ONE.

[14] Liu H., Antony S., Roy K., Juhasz A., Wu Y., Lu J., Meitzler J.L., Jiang G., Polley E., Doroshow J.H. (2017). Interleukin-4 and interleukin-13 increase NADPH oxidase 1-related proliferation of human colon cancer cells. Oncotarget.

[15] Sharma P., Chakraborty R., Wang L., Min B., Tremblay M.L., Kawahara T., Lambeth J.D., Haque S.J. (2008). Redox regulation of interleukin-4 signaling. Immunity.

[16] Halasi M., Wang M., Chavan T.S., Gaponenko V., Hay N., Gartel A.L. (2013). ROS inhibitor *N*-acetyl L-cysteine antagonizes the activity of proteasome inhibitors. Biochem J..

[17] Bur H., Haapasaari K.M., Turpeenniemi-Hujanen T., Kuittinen O., Auvinen P., Marin K., Koivunen P., Sormunen R., Soini Y., Karihtala P. (2014). Oxidative stress markers and mitochondrial antioxidant enzyme expression are increased in aggressive Hodgkin lymphomas. Histopathology.

[18] Giefing M., Winoto-Morbach S., Sosna J., Döring C., Klapper W., Küppers R., Böttcher S., Adam D., Siebert R., Schütze S. (2013). Hodgkin-Reed-Sternberg cells in classical Hodgkin lymphoma show alterations of genes encoding the NADPH oxidase complex and impaired reactive oxygen species synthesis capacity. PLoS ONE.

[19] Brune V., Tiacci E., Pfeil I., Döring C., Eckerle S., van Noesel C.J., Klapper W., Falini B., von Heydebreck A., Metzler D. (2008). Origin and pathogenesis of nodular lymphocyte-predominant Hodgkin lymphoma as revealed by global gene expression analysis. J. Exp. Med..

[20] Stuible M., Doody K.M., Tremblay M.L. (2008). PTP1B and TC-PTP: Regulators of transformation and tumorigenesis. Cancer Metastasis Rev..

[21] Burdon R.H. (1996). Control of cell proliferation by reactive oxygen species. Biochem. Soc. Trans..

[22] Burdon R.H., Gill V., Alliangana D. (1996). Hydrogen peroxide in relation to proliferation and apoptosis in BHK-21 hamster fibroblasts. Free Radic. Res..

[23] Burdon R.H., Gill V., Boyd P.A., Rahim R.A. (1996). Hydrogen peroxide and sequence-specific DNA damage in human cells. FEBS Lett..

[24] Skinnider B.F., Elia A.J., Gascoyne R.D., Patterson B., Trumper L., Kapp U., Mak T.W. (2002). Signal transducer and activator of transcription 6 is frequently activated in Hodgkin and Reed-Sternberg cells of Hodgkin lymphoma. Blood.

[25] Zeng M., Chen Y., Jia X., Liu Y. (2020). The Anti-Apoptotic Role of EBV-LMP1 in Lymphoma Cells. Cancer Manag. Res..

[26] Bánfi B., Clark R.A., Steger K., Krause K.-H. (2003). Two novel proteins activate superoxide generation by the NADPH oxidase NOX1. J. Biol. Chem..

[27] Gough N.R. (2008). Calcium Signals Prime Macrophages. Sci. Signal..

[28] Trümpler A., Schlott B., Herrlich P., Greer P.A., Böhmer F. (2009). Calpain-mediated degradation of reversibly oxidized protein-tyrosine phosphatase 1B. FEBS J..

[29] Boivin B., Yang M., Tonks N.K. (2010). Targeting the reversibly oxidized protein tyrosine phosphatase superfamily. Sci. Signal..

[30] Tonks N.K. (2005). Redox redux: Revisiting PTPs and the control of cell signaling. Cell.

[31] Konaté M.M., Antony S., Doroshow J.H. (2020). Inhibiting the Activity of NADPH Oxidase in Cancer. Antioxid. Redox Signal..

[32] den Hertog J., Groen A., van der Wijk T. (2005). Redox regulation of protein-tyrosine phosphatases. Arch Biochem. Biophys.

[33] Meng F.G., Zhang Z.Y. (2013). Redox regulation of protein tyrosine phosphatase activity by hydroxyl radical. Biochim. Biophys Acta..

[34] Zahn M., Kaluszniak B., Möller P., Marienfeld R. (2021). The PTP1B mutant PTP1B∆2-4 is a positive regulator of the JAK/STAT signalling pathway in Hodgkin lymphoma. Carcinogenesis.

[35] Chen C., Kong A.N. (2005). Dietary cancer-chemopreventive compounds: From signaling and gene expression to pharmacological effects. Trends Pharmacol. Sci..

[36] Liu X.M., Azam M.A., Peyton K.J., Ensenat D., Keswani A.N., Wang H., Durante W. (2007). Butylated hydroxyanisole stimulates heme oxygenase-1 gene expression and inhibits neointima formation in rat arteries. Cardiovasc. Res..

[37] Song J.D., Kim K.M., Kim K.H., Kim C.D., Kim J.M., Yoo Y.H., Park Y.C. (2008). Differential role of diphenyleneiodonium, a flavoenzyme inhibitor, on p53-dependent and -independent cell cycle progression. Int. J. Oncol..

[38] Yuan X., Xu C., Pan Z., Keum Y.-S., Kim J.-H., Shen G., Yu S., Oo K.T., Ma J., Kong A.-N.T. (2006). Butylated hy-droxyanisole regulates ARE-mediated gene expression via Nrf2 coupled with ERK and JNK signaling pathway in HepG2 cells. Mol. Carcinog..

[39] Huang L., Liu D., Wang N., Ling S., Tang Y., Wu J., Hao L., Luo H., Hu X., Sheng L. (2018). Integrated genomic analysis identifies deregulated JAK/STAT-MYC-biosynthesis axis in aggressive NK-cell leukemia. Cell Res..

[40] Ding B.B., Yu J.J., Yu R.Y.-L., Mendez L.M., Shaknovich R., Zhang Y., Cattoretti G., Ye B.H. (2008). Constitutively activated STAT3 promotes cell proliferation and survival in the activated B-cell subtype of diffuse large B-cell lymphomas. Blood.

[41] Song L., Rawal B., Nemeth J.A., Haura E.B. (2011). JAK1 activates STAT3 activity in non-small-cell lung cancer cells and IL-6 neu-tralizing antibodies can suppress JAK1-STAT3 signaling. Mol. Cancer Ther..

[42] Wang J., Lv X., Guo X., Dong Y., Peng P., Huang F., Wang P., Zhang H., Zhou J., Wang Y. (2021). Feedback ac-tivation of STAT3 limits the response to PI3K/AKT/mTOR inhibitors in PTEN-deficient cancer cells. Oncogenesis.

