# Supplementary material for: Intracellular Oxidant Levels Are Crucial for Cell Survival and JAK/STAT Signaling in Classical Hodgkin’s Lymphoma
Source: Antioxidants (Basel). 2026 Jan 9;15(1):90. doi: 10.3390/antiox15010090 (PMC12838041; doi:10.3390/antiox15010090)
Supplement: Supplementary file 1 [file antioxidants-15-00090-s001.zip › antioxidants-4029868-supplementary.pdf]

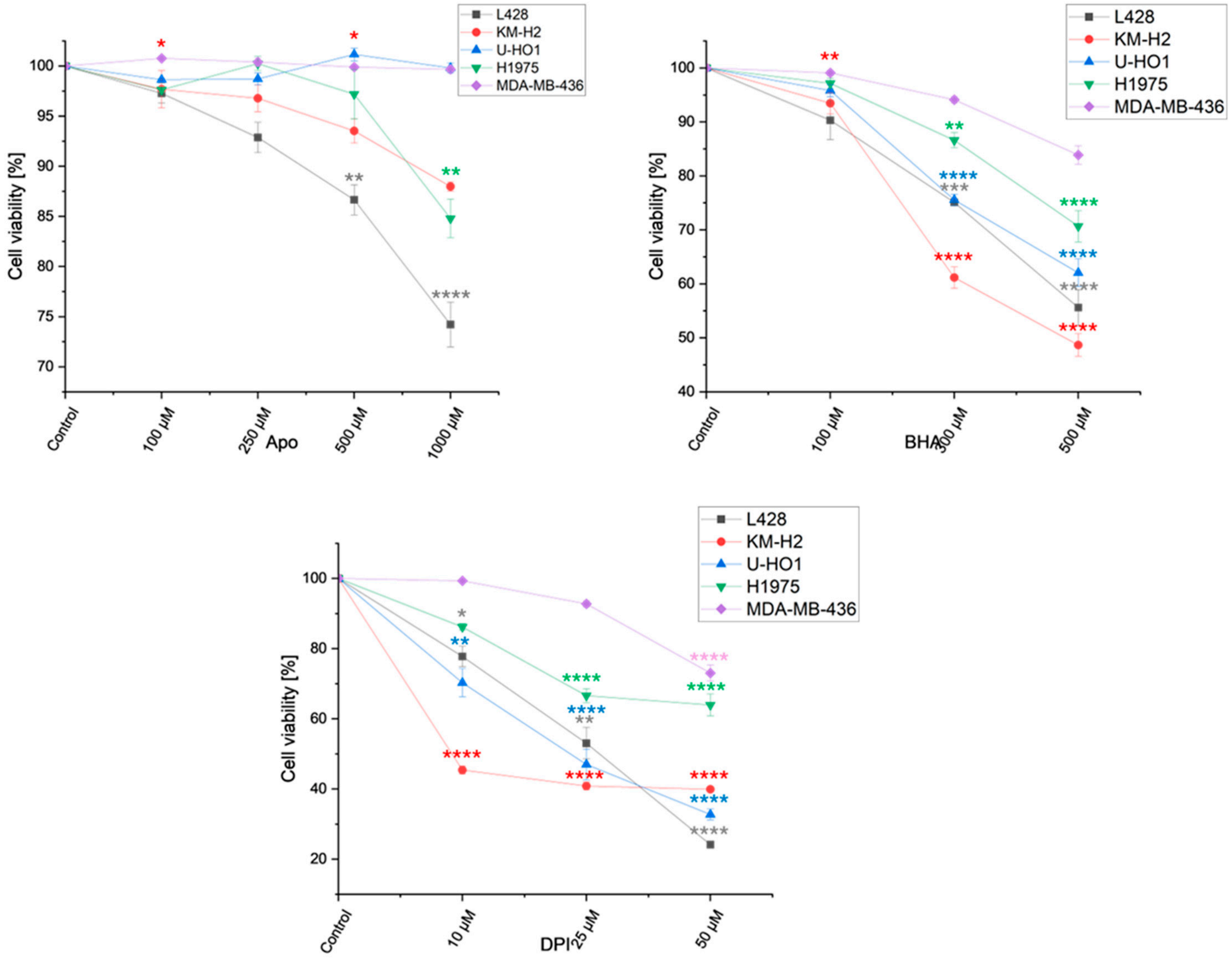

Supplementary Figure S1. Treatment with Apo, BHA, or DPI affects cell viability. To assess cell viability, L428, KM-H2, U-HO1, H1975, and MDA-MB-436 cells were seeded at a density of >600.000 cells/ml and treated for 48 hours with various concentrations of DMSO, apocynin, BHA, or DPI. Total cell number and percentage of viable cells were determined using the ViCell counting system (n=3). *p < 0.05; **p < 0.01; ***p < 0.001; ****p < 0.0001

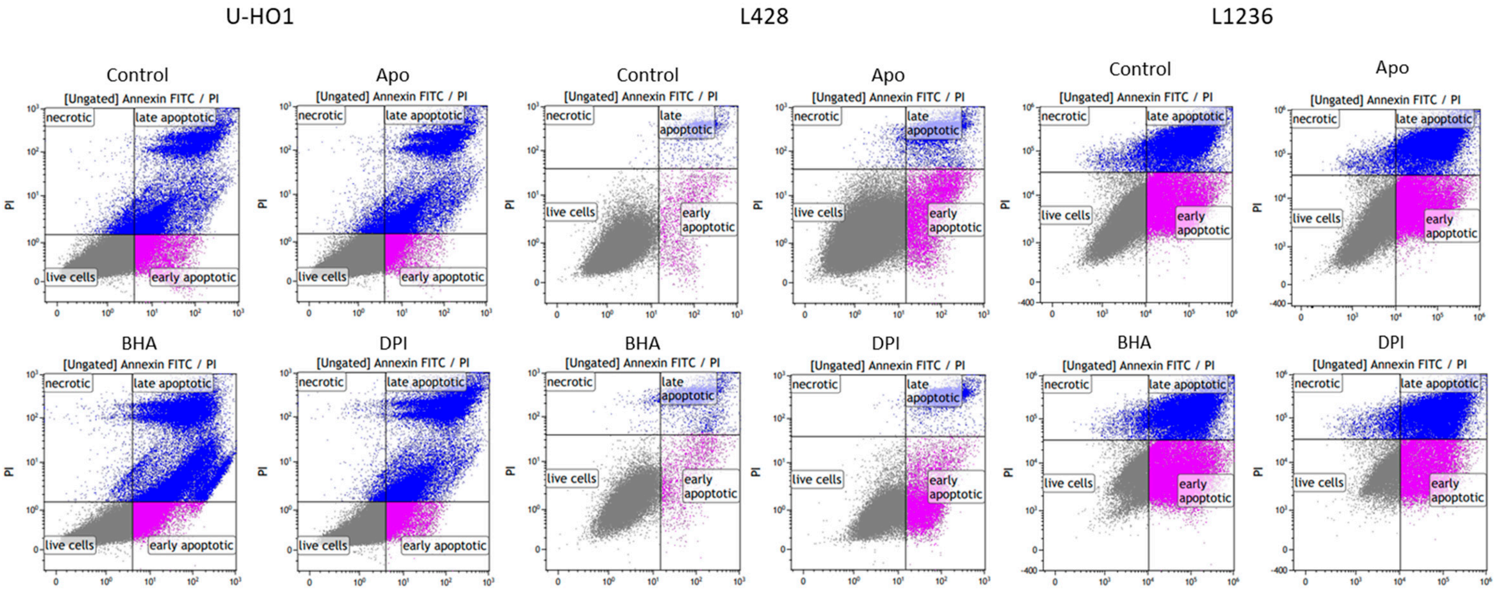

Supplementary Figure S2. Quantification of cell death reveals distinct populations of live, early apoptotic, late apoptotic, and necrotic cells in cHL cell lines following Apo, BHA, or DPI treatment. Cells were treated for 24 hours with DMSO, Apo (500 µM), BHA (300 µM), or DPI (25 µM). Apoptotic and necrotic cells were quantified by Annexin V/PI staining followed by flow cytometry analysis.

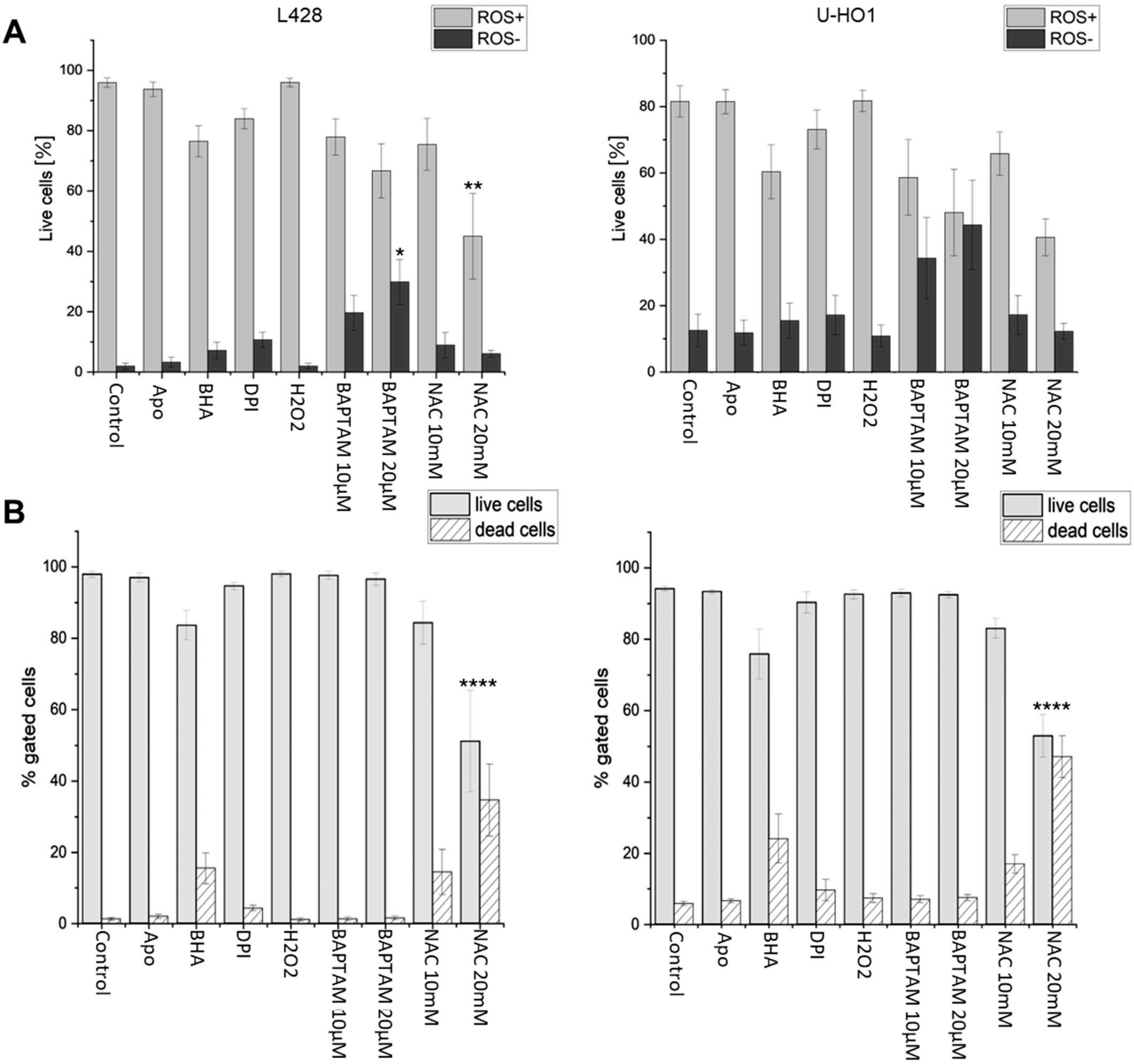

Supplementary Figure S3. Determination of ROS levels and cell viability in cHL cell lines following Apo, BHA, or DPI treatment. Cells were treated for 24 hours with DMSO, Apo (500 µM), BHA (300 µM), or DPI (25 µM) as well as BAPTA-AM and NAC for 1 hour, and 10 µM H_2O_2 for 30 minutes. A: Ratio of oxidant -positive and oxidant-negative cells among living cells. B: Ratio between living and dead cells. Intracellular oxidant (ROS) levels were quantified using H_2DCFDA/PI staining followed by flow cytometric analysis. In parallel, the proportions of viable and non-viable cells were determined (*n*=4). *$p < 0.05$; **$p < 0.01$; ****$p < 0.0001$

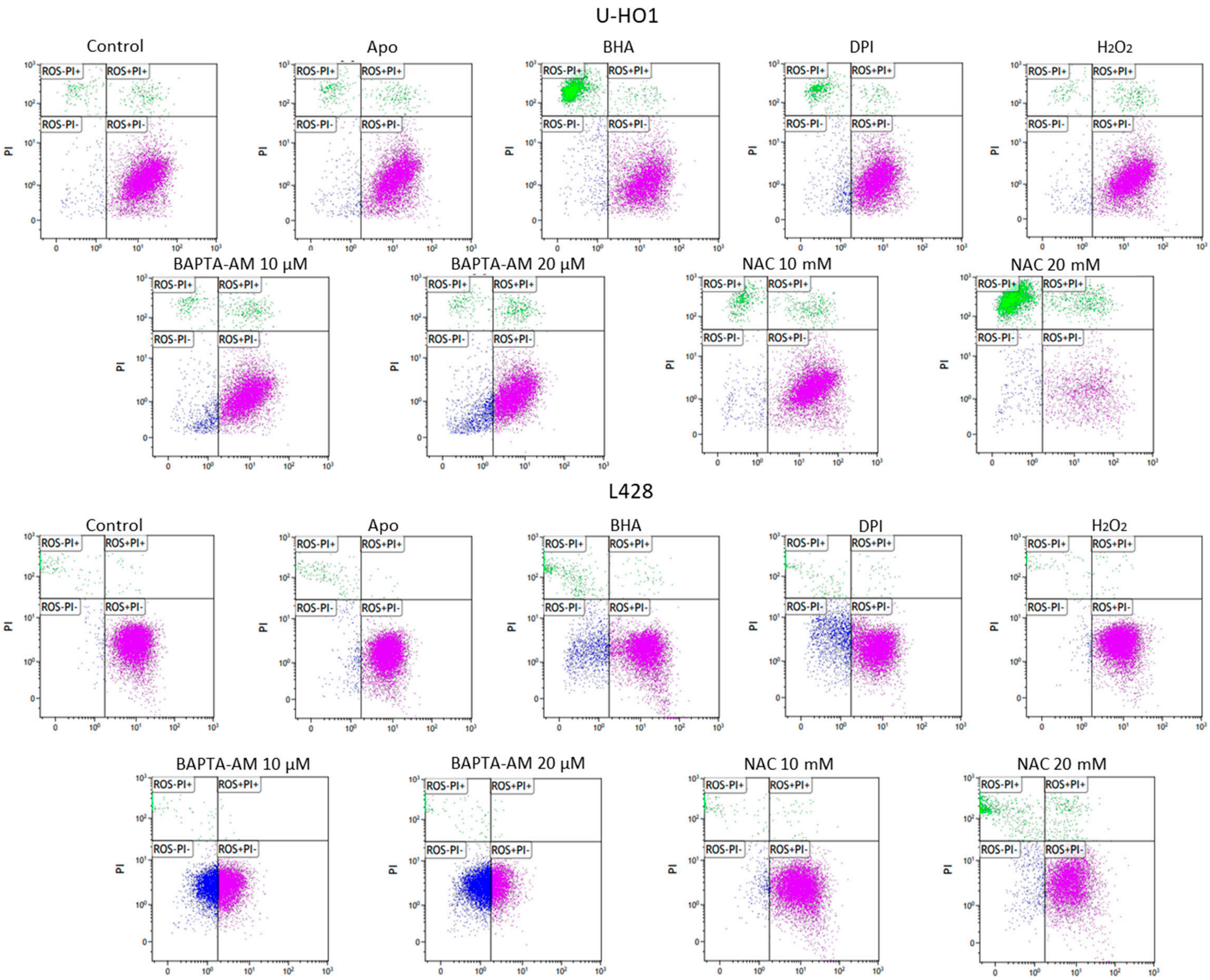

Supplementary Figure S4. Quantification of intracellular oxidant levels (ROS) in cHL cell lines following Apo, BHA, or DPI treatment. Cells were treated for 24 hours with DMSO, Apo (500 µM), BHA (300 µM), or DPI (25 µM) as well as BAPTA-AM and NAC for 1 hour, and 10 µM H_2O_2 for 30 minutes. Intracellular oxidant levels were quantified using H_2DCFDA/PI staining followed by flow cytometric analysis.

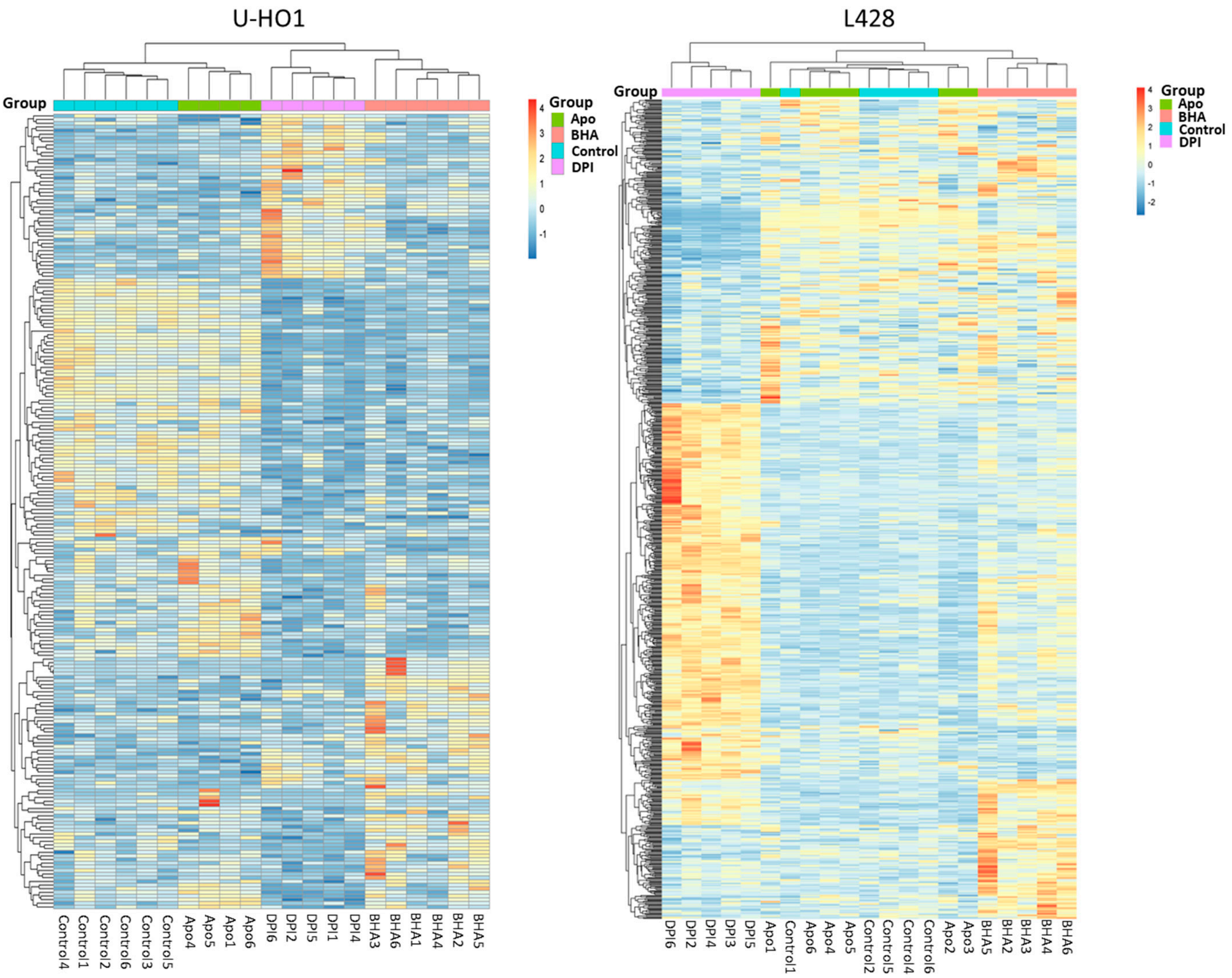

Supplementary Figure S5. Unsupervised clustering of differentially expressed genes in L428 and U-HO1 cells after Apo, BHA, or DPI treatment. This heatmap shows unsupervised clustering of differentially expressed genes in L428 and U-HO1 cells treated for 24 hrs with DMSO, Apo (250 μM), BHA (200 μM), or DPI (25 μM). The top color bar indicates the respective treatments. Differential expression analysis was performed using a false discovery rate (FDR) cutoff of < 0.05 and a log fold change (logFC) threshold of 1.

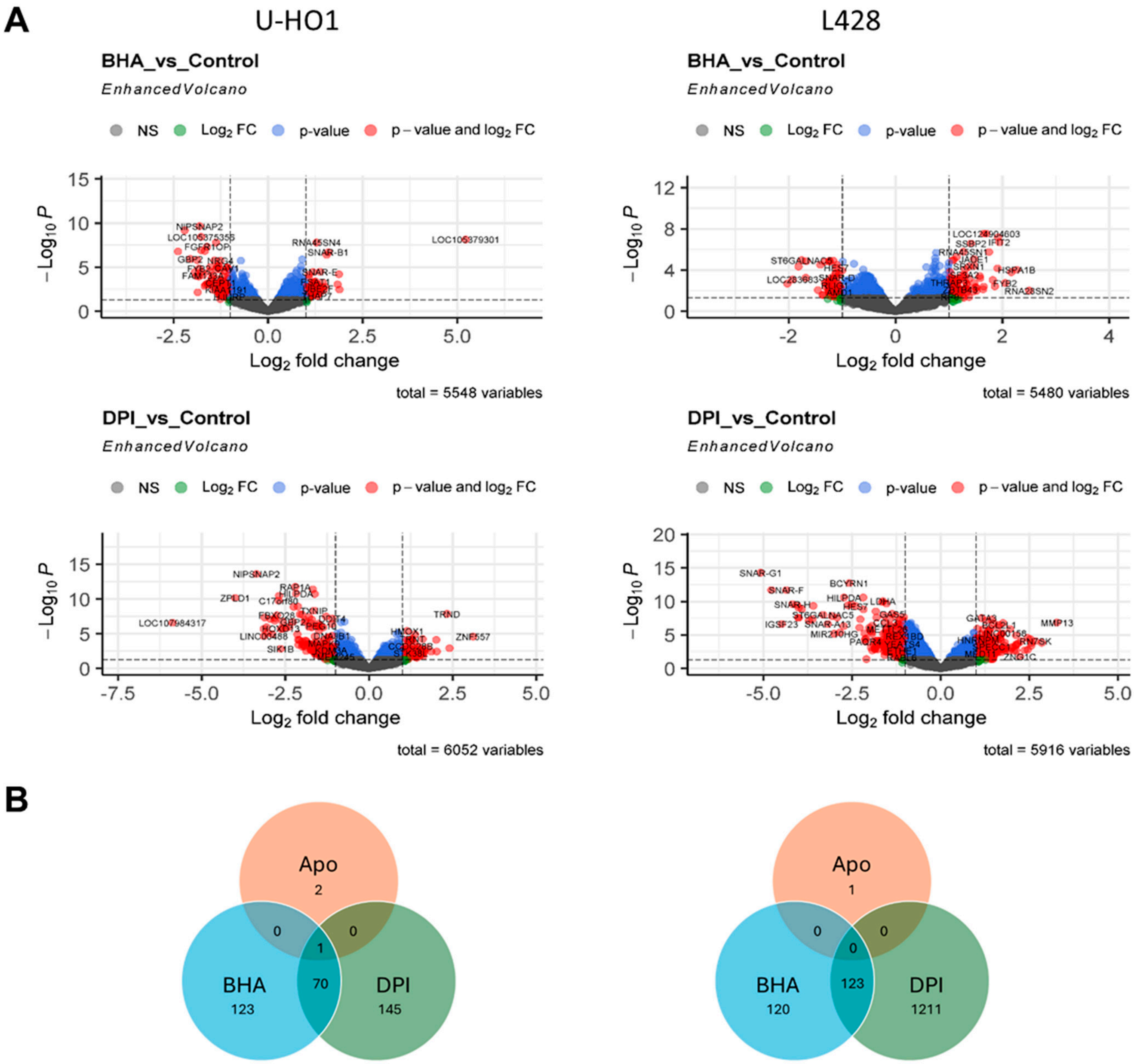

Supplementary Figure S6. BHA and DPI treatment induce significant gene expression in L428 and U-HO1 cells. L428 and U-HO1 cells were treated for 24 hrs with DMSO, Apo (250 μM), BHA (200 μM), or DPI (25 μM). *A*: Volcano plots illustrate significantly up- and downregulated genes in L428 and U-HO1 cells treated with BHA and DPI, compared to DMSO control. *B*: Venn diagrams showing the overlap of differentially expressed genes among the three treatments, based on an absolute fold change ≥ 1 and an FDR-adjusted p-value < 0.1. Venn diagrams were generated using GeneGlobe (Qiagen).

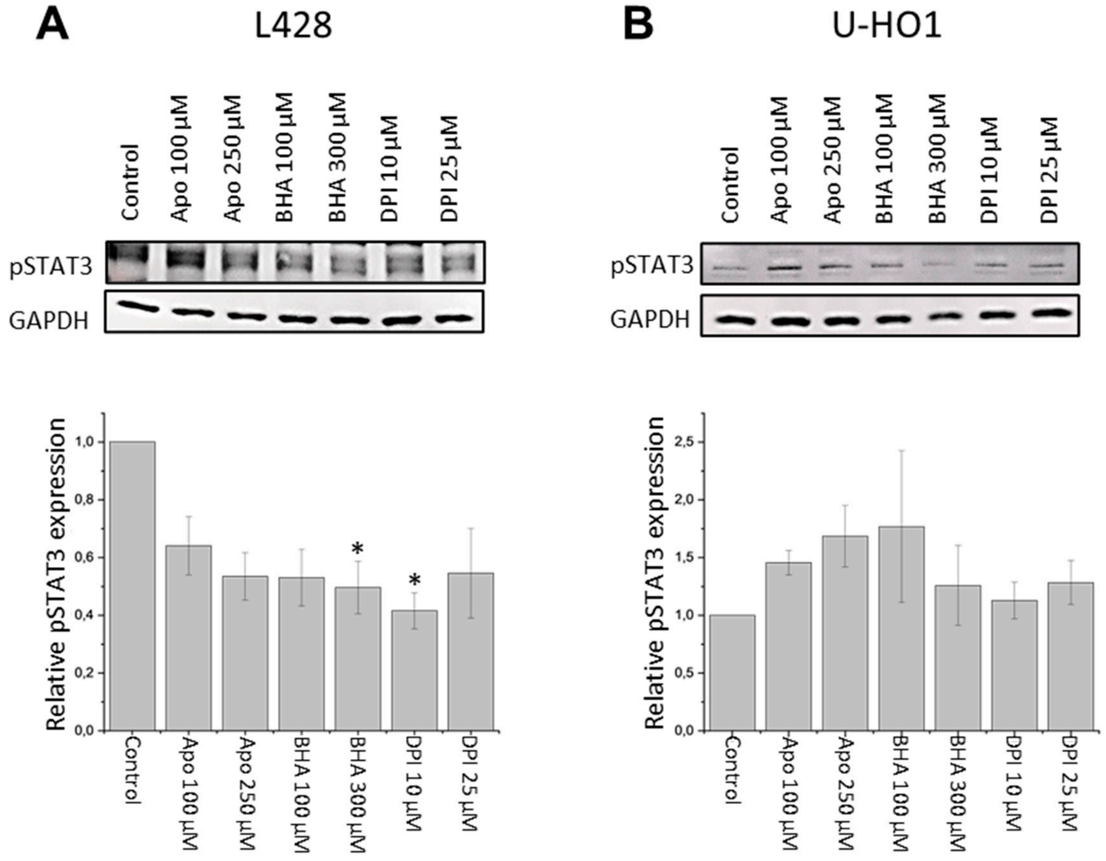

Supplementary Figure S7. Treatment with Apo, BHA, and DPI attenuates pSTAT3 signaling. A: Immunoblot analysis of whole cell extracts from the cHL cell line L428 using antibodies specific for pSTAT3 and GAPDH as loading control. B: Immunoblot analysis of whole cell extracts from the cHL cell line U-HO1 using antibodies specific for pSTAT3 and GAPDH as loading control. Cells were treated with DMSO, Apo, BHA, or DPI, as indicated for 24 h. L428 n=4; U-HO1 n=3; *$p < 0.05$

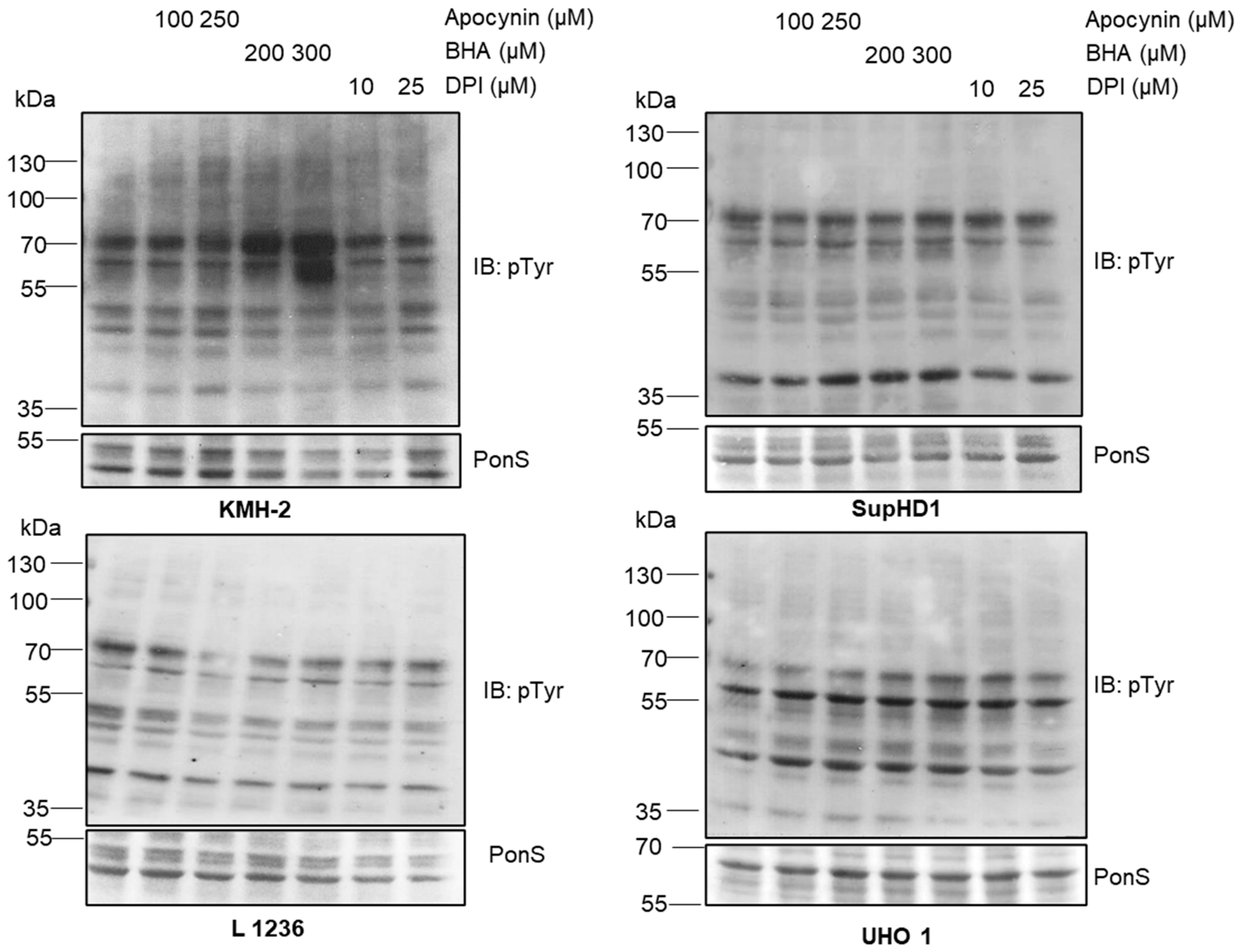

Supplementary Figure S8. Global tyrosine phosphorylation (pTyr) profiles in cHL cell lines are not altered by Apo, BHA, or DPI treatment. Western blot analysis of whole cell extracts from the indicated cHL cell lines using an antibody specific to pTyr. Equal protein loading was verified by PonceauS (PonS) staining of the membrane. Cells were treated with DMSO as control, apocynin, BHA, or DPI for 24 hrs.

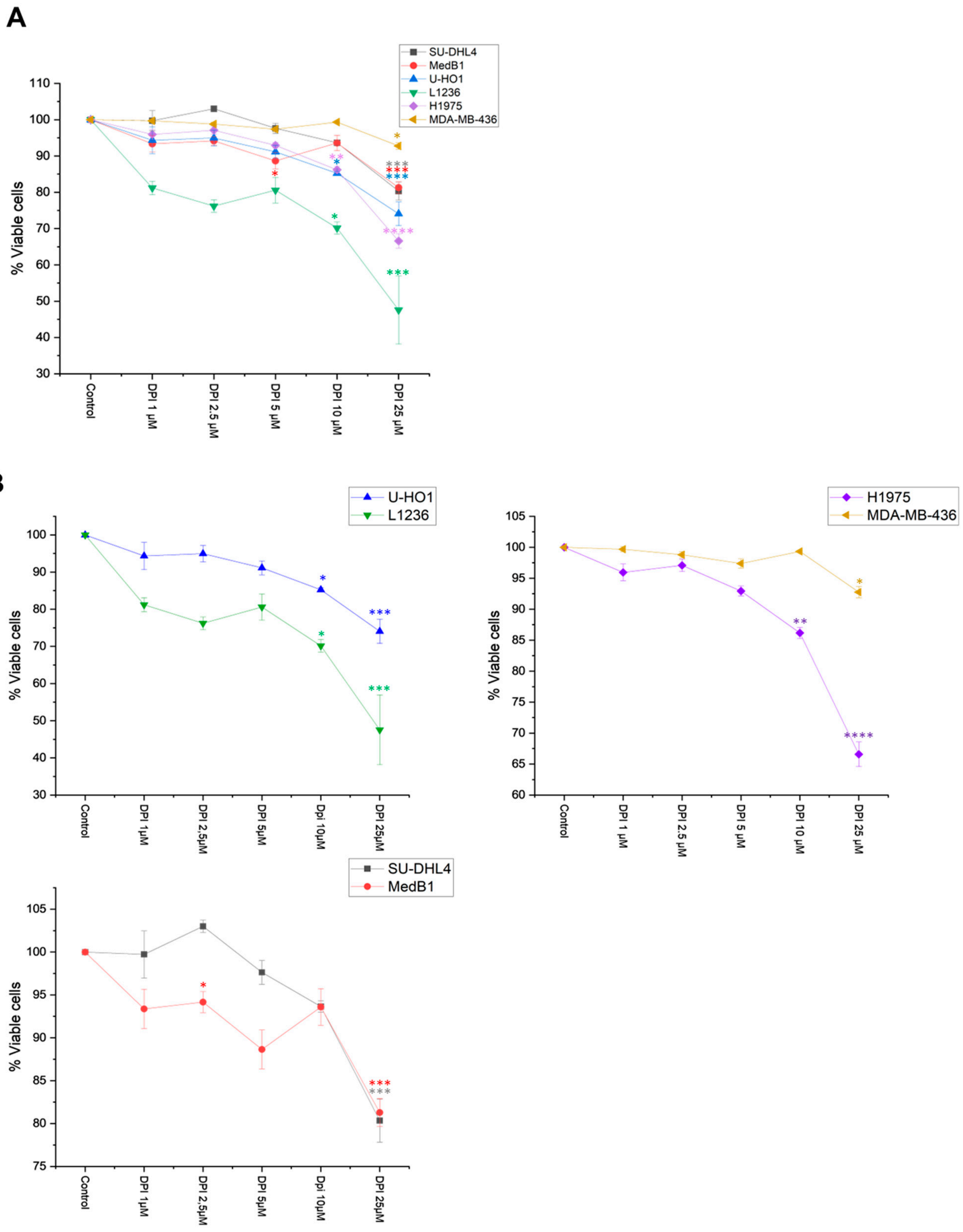

Supplementary Figure S9. DPI treatment affects cell viability differently across cell types. To assess cell viability, SU-DHL4, Med-B1, U-HO1, L1236, H1975, and MDA-MB-436 cells were seeded at a density of >600.000 cells/ml and treated for 48 hours with various concentrations of DPI. Total cell number and percentage of viable cells were determined using the ViCell counting system (n=3). *$p < 0.05$; **$p < 0.01$; ***$p < 0.001$; ****$p < 0.0001$
